# Hybrid Ginseng‐derived Extracellular Vesicles‐Like Particles with Autologous Tumor Cell Membrane for Personalized Vaccination to Inhibit Tumor Recurrence and Metastasis

**DOI:** 10.1002/advs.202308235

**Published:** 2024-02-14

**Authors:** Haoran Wang, Jiankang Mu, Yexing Chen, Yali Liu, Xianghui Li, Hao Li, Peng Cao

**Affiliations:** ^1^ Jiangsu Provincial Medical Innovation Center Affiliated Hospital of Integrated Traditional Chinese and Western Medicine Nanjing University of Chinese Medicine Nanjing 210028 China; ^2^ School of Pharmacy Nanjing University of Chinese Medicine Nanjing 210023 China; ^3^ Department of Dermatology The First Affiliated Hospital of Guangxi Medical University Nanning 530021 China; ^4^ Chinatalentgroup (CTG Group) Beijing 100020 China

**Keywords:** ginseng‐derived nanoparticles, hybrid nanoparticles, long‐term protection, mature DCs, personalized tumor vaccine

## Abstract

Personalized cancer vaccines based on resected tumors from patients is promising to address tumor heterogeneity to inhibit tumor recurrence or metastasis. However, it remains challenge to elicit immune activation due to the weak immunogenicity of autologous tumor antigens. Here, a hybrid membrane cancer vaccine is successfully constructed by membrane fusion to enhance adaptive immune response and amplify personalized immunotherapy, which formed a codelivery system for autologous tumor antigens and immune adjuvants. Briefly, the functional hybrid vesicles (HM‐NPs) are formed by hybridizing ginseng‐derived extracellular vesicles‐like particles (G‐EVLPs) with the membrane originated from the resected autologous tumors. The introduction of G‐EVLPs can enhance the phagocytosis of autologous tumor antigens by dendritic cells (DCs) and facilitate DCs maturation through TLR4, ultimately activating tumor‐specific cytotoxic T lymphocytes (CTLs). HM‐NPs can indeed strengthen specific immune responses to suppress tumors recurrence and metastasis including subcutaneous tumors and orthotopic tumors. Furthermore, a long‐term immune protection can be obtained after vaccinating with HM‐NPs, and prolonging the survival of animals. Overall, this personalized hybrid autologous tumor vaccine based on G‐EVLPs provides the possibility of mitigating tumor recurrence and metastasis after surgery while maintaining good biocompatibility.

## Graphical abstract

Personalized cancer treatment continues to evolve, driven by the pursuit of more precise and effective therapies. Here, a plant‐based hybrid personalized tumor vaccine forms a co‐delivery system for personalized tumor antigens and adjuvants aiming to amend long‐term immune protection induced by autologous tumor antigens against tumor recurrence and metastasis after surgery.

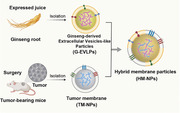

## 1 Introduction

The imperative to develop potent anti‐tumor strategy has become increasingly urgent,^[^ ^1^ ^]^ with the goal of eradicating the recurrence of solid tumors and disseminated metastasis after surgical interventions.^[^ ^2^ ^]^ Personalized cancer vaccine, which triggers tumor‐specific cell‐mediated immunity to eliminate tumors, stands out as a particularly promising approach in providing long‐term protection.^[^ ^3^ ^]^ Since surgery remains the primary choice for the majority of solid tumors, utilizing antigens from resected tumors directly to create a personalized cancer vaccine seems uncomplicated and cost‐saving.^[^ ^4^ ^]^ However, this therapeutic approach showed limited clinical benefit, which is mainly attributed to the challenge in initiating a robust antitumor immune response for the weak immunogenicity of tumor antigens.^[^ ^5^ ^]^ Hence, an appropriate vaccine delivery system to convey autologous tumor antigens is required, aiming to bolster the initiation of a more robust immune response and improve treatment outcomes in clinic.^[^ ^6^ ^]^

Due to the natural advantages, derivatives of plants have been coupled with standard tumor therapies, and have achieved improved efficacy.^[^ ^7^ ^]^ Vesicles derived from plants have gained recognition for their predominant pharmacological and immunomodulatory capabilities, and can be easily modified.^[^ ^8^ ^]^ Especially, plants‐derived nanovesicles can also be used as drug delivery carrier to further enhance the therapeutic effect on diseases while maintaining the original characteristics.^[^ ^9^ ^]^ For cancer vaccines, vesicles with pro‐inflammatory activity can be exploited to act as vaccine delivery platform to enhance specific antitumor reactions by promoting adaptive immune responses.^[^ ^10^ ^]^ Our previous studies found that ginseng‐derived extracellular vesicles‐like particles (G‐EVLPs) extracted from fresh ginseng roots have good ability to be internalized by monocytes and activate monocytes through TLR4.^[^ ^11^ ^]^ Thence, the unexplored potential of G‐EVLPs as a foundation for personalized cancer vaccines could be delved into by establishing a co‐delivery formulation based on autologous tumor antigens and G‐EVLPs as adjuvants.

**Figure 1:**
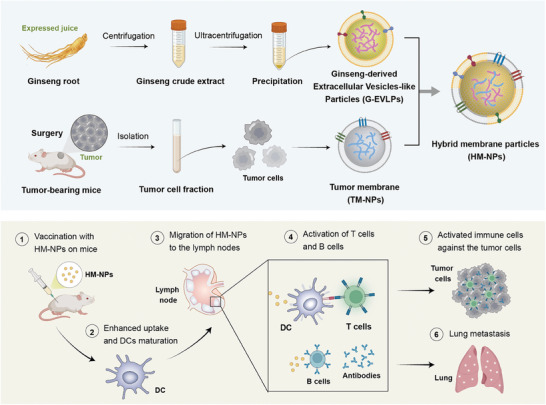
Schematic illustration of the construction and mechanistic process of the cancer vaccine HM‐NPs. a) The preparation procedure of the HM‐NPs. TM‐NPs were obtained from surgically removed tumors on tumor‐bearing mice. Ginseng roots were squeezed to obtain ginseng fluid, and the extraction process was used to prepare G‐EVLPs. Using an extruder, the cancer vaccine HM‐NPs were generated by extruding the mixed G‐EVLPs and TM‐NPs. b) The mechanistic process of HM‐NPs. ① HM‐NPs were subcutaneous injected at the base of tail. ② HM‐NPs enhanced uptake of tumor membrane antigen and dendritic cells (DCs) maturation. ③ DCs migrated to the inguinal lymph nodes after uptake of tumor membrane antigen and maturation. ④ HM‐NPs elicited specific cytotoxic T lymphocytes and B lymphocytes, and formed strengthened anti‐tumor immune memory. ⑤ and ⑥ HM‐NPs activated enhanced personalized adaptive immune response to inhibit the tumor recurrence and tumor metastasis after subcutaneous injection.

Recently, the separated tumor cell membrane from primary tumors was recognized as another personalized tumor vaccine candidate for covering a high level of antigenic motifs.^[^ ^12^ ^]^ Nevertheless, the weak immunogenicity of tumor antigens and the immunosuppressive signals on cell membrane synergistically suppress immune recognition.^[^ ^1^, ^13^ ^]^ Therefore, the G‐EVLPs might have the potential to enhance the immunogenicity of the tumor cell and strengthen immune recognition. To this end, here, we developed the hybrid membrane nanoparticles vaccine (HM‐NPs) formed by hybridizing G‐EVLPs with the autologous tumor membrane from resected tumors to boost the immune responses for personalized immunotherapy (**Figure** **1**). This personalized vaccine can enhance the phagocytosis of autologous tumor antigens by dendritic cells (DCs) and facilitate DCs maturation through TLR4,^[^ ^14^ ^]^ and build an efficient immune protection to prolong the survival while maintaining good biocompatibility. The immune activation properties and the vaccine activity in various recurrent and/or metastatic mouse animal models of HM‐NPs was simultaneously investigated to highlight the clinical translation potential.

## 2 Result

### 2.1 HM‐NPs Possess The Properties of Both GdNPs and TM‐NPs with A Typical Spherical Structure

**Figure 2:**
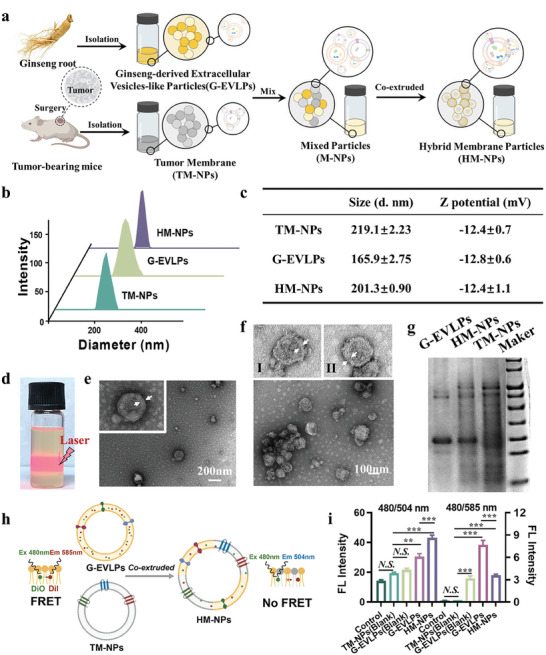
Construction and characterization of the cancer vaccine HM‐NPs (B16F10). a) Schematic figure of the manufacture process of the cancer vaccine HM‐NPs. b) Particle size distribution of TM‐NPs, G‐EVLPs and HM‐NPs determined by DLS. c) Particle size and ζ potential of TM‐NPs, G‐EVLPs and HM‐NPs (n = 3). d) Tyndall phenomenon of HM‐NPs with 660 nm laser irradiation on the right. e) Transmission electron microscope (TEM) images of G‐EVLPs (Insert image. enlarged TEM figure of G‐EVLPs. The white arrows indicate the bilayer lipid membrane). f) Transmission electron microscope (TEM) images of HM‐NPs (Insert image I. enlarged TEM figure of HM‐NPs. Insert figure II. enlarged TEM figure of HM‐NPs. The white arrows indicate the bilayer lipid membrane). g) SDS‐PAGE protein analysis of Marker, G‐EVLPs, TM‐NPs and HM‐NPs. h) FRET‐based lipid mixing assay used to monitor the fusion between G‐EVLPs and TMNPs. i) Fluorescence intensity of TM‐NPs (Blank), G‐EVLPs (Blank), G‐EVLPs (with fluorescent dyes) and HM‐NPs (with fluorescent dyes) at Ex/Em = 480 nm/504 nm and Ex/Em = 480 nm/585 nm (n = 3). Data are representative or pooled and are expressed as Mean ± SE. Asterisks indicate statistically significant differences as analyzed by One‐Way ANOVA (∗∗∗*p* < 0.001, ∗∗*p* < 0.01, N.S. *p* > 0.05).

To obtain a personalized tumor vaccine hybridized with G‐EVLPs, we first needed to prepare HM‐NPs. The processing of HM‐NPs was mainly obtained through the following three steps: (I) isolation of fresh ginseng roots G‐EVLPs as described previously,^[^ ^11a^ ^]^ (II) preparation of the TM‐NPs from surgically resected tumors, (III) fusion of G‐EVLPs and TM‐NPs to prepare HM‐NPs, M‐NPs formed by simple mixing without extrusion (**Figure** **2**a). HM‐NPs were obtained by fusing the G‐EVLPs and TM‐NPs at protein mass ratios of 1:5, 3:5, 5:5 repeatedly using the extruder through a porous polycarbonate membrane (200 nm and 400 nm). To determine an appropriate ratio to promote DCs uptake and maturation, the obtained HM‐NPs were co‐incubated with bone marrow‐derived DCs (BMDCs) for 24 h to detect the ratio of CD11c^+^CD80^+^CD86^+^ cells, and TLR4 agonist lipopolysaccharide (LPS, 50 µg mL^−1^) was used as positive control drug. The group with a protein ratio of 1:5 (G‐EVLPs:TM‐NPs) could already provoke the maturation of BMDCs to a high degree (Figure S1, Supporting Information), and we chose the 1:5 protein mass ratio for the subsequent experiments.

Dynamic light scattering (DLS) analysis indicated that the HM‐NPs were about 200 nm in diameter (Figure 2b), which is similar to TM‐NPs and G‐EVLPs. The surface ζ potential of HM‐NPs was −12.4 mV at the protein ratio of 1:5 (G‐EVLPs:TM‐NPs), which is also similar to TM‐NPs and G‐EVLPs (Figure 2c). Meanwhile, HM‐NPs should exhibit obvious Tyndall phenomenon upon laser incidence in this size range, which was confirmed by the follow‐up experimental results (Figure 2d). To demonstrate that the as‐prepared HM‐NPs could possess vesicle‐like characteristics, we characterized the HM‐NPs using transmission electron microscopy (TEM). TEM images showed that HM‐NPs exhibited a uniform spherical nanostructure with the structure of bilayer lipid membrane (Figure 2f), which was consistent with the morphology of G‐EVLPs obtained previously (Figure 2e). At the same time, Cryo‐SEM images also demonstrated that HM‐NPs have a distinct spherical structure (Figure S2, Supporting Information).

Using the membrane fusion method, we found that a variety of tumor cells including 4T1 and MB49 can obtain the corresponding HM‐NPs (Figure S3, Supporting Information). Sodium dodecyl sulfonate‐polyacrylamide gel electrophoresis (SDS‐PAGE) was adopted to prove that HM‐NPs possess the characteristics of both G‐EVLPs and TM‐NPs. The proteomic profiles were examined, and HM‐NPs indeed presented the corresponding protein bands of G‐EVLPs and TM‐NPs (Figure 2g; Figure S4, Supporting Information). Hereafter, to prove that the prepared HM‐NPs were indeed obtained through membrane fusion, we used the fluorescence resonance energy transfer (FRET) to verify (Figure 2h).^[^ ^15^ ^]^ We used DiO and DiI to simultaneously label G‐EVLPs. After fusion with TM‐NPs membrane to obtain HM‐NPs, we found that the fluorescence intensity became stronger at Ex/Em = 480/504 nm, while the fluorescence intensity decreased at Ex/Em = 480/585 nm (Figure 2i). At the same time, we used flow cytometry to determine the fusion efficiency of HM‐NPs (59.6%) by analyzing the proportion of DiD and DiR double‐positive particles. This result indicated that the fusion process of the lipid membrane occurred. Furthermore, the obtained HM‐NPs remained stable for 2 weeks when stored at 4 °C.

### 2.2 HM‐NPs Enhance Tumor Antigen Uptake and Activation of BMDCs In Vitro

**Figure 3:**
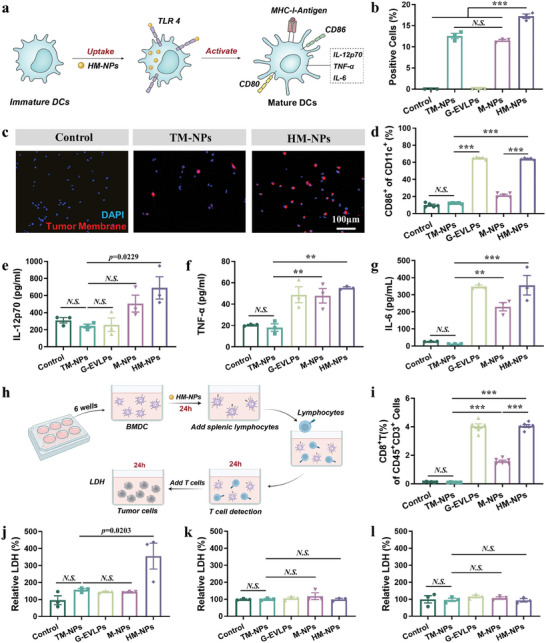
HM‐NPs (B16F10) enhance tumor antigen uptake and activation of immune cells in vitro. a) Schematic illustration of the BMDCs uptake and activation experiments. b) Cellular uptake of DiD‐labeled tumor membrane after a 24‐hour incubation with BMDCs, as assessed by flow cytometry. c) Confocal images of BMDCs for TM‐NPs and HM‐NPs uptake after a 24‐hour incubation (DiD‐labeled tumor membrane). d) Flow cytometry analysis of CD11c^+^CD86^+^ BMDCs after incubation with different NPs for 24 hours. e) Secretion of IL‐12p70 in supernatant of the BMDCs medium measured by ELISA kit. f) Secretion of TNF‐α in supernatant of the BMDCs medium measured by ELISA kit. g) Secretion of IL‐6 in supernatant of the BMDCs medium measured by ELISA kit. h) Schematic illustration of the specific immune activation experiments in vitro. i) Percentage of splenic CD8^+^ T lymphocytes activation. j‐l. LDH concentration in the supernatant after co‐incubation of the splenic T lymphocytes with B16F10 tumor cells j), CT26 tumor cells k) and 4T1 tumor cells l). Data are representative or pooled and are expressed as Mean ± SE. Asterisks indicate statistically significant differences as analyzed by One‐Way ANOVA (∗∗∗*p* < 0.001, ∗∗*p* < 0.01, N.S. *p* > 0.05).

The purpose of the study is to prepare HM‐NPs to promote the uptake of tumor‐associate antigens by DCs and promote the maturation of DCs, and enhance the activation of the immune system and personalized immunotherapy (**Figure** **3**a). Internalization of tumor‐associate antigens into DCs is the first step for processing and presentation.^[^ ^16^ ^]^ Thus, we first detected the internalization of DiD‐labeled TM‐NPs (derived from B16F10 cells) by BMDCs (Figure 3a). Using flow cytometry, we assessed the results of internalization of TM‐NPs, M‐NPs and HM‐NPs by BMDCs after incubating for 24 h (M‐NPs: simply mixed the G‐EVLPs and TM‐NPs). The results proved that the internalization of HM‐NPs by BMDCs was much higher than that of TM‐NPs (Figure 3b; Figure S5, Supporting Information). The results of confocal fluorescence imaging could further illustrate the point, and formation of HM‐NPs did increase the co‐localization of antigens (tumor membrane) and adjuvant (G‐EVLPs) in BMDCs (Figure 3c; Figure S6, Supporting Information). Together, these results indicated that the HM‐NPs obtained by fusion with G‐EVLPs can indeed increase the uptake of tumor‐associated antigens by BMDCs.

Toll‐like receptors (TLRs) recognize pathogen‐associated molecular patterns (PAMPs) to initiate immune responses.^[^ ^17^ ^]^ Our previous studies showed that G‐EVLPs can activate monocytes through TLR4.^[^ ^11b^ ^]^ To prove that the fused HM‐NPs can still activate BMDCs, we detected the proportion of mature BMDCs by flow cytometry. We found that HM‐NPs significantly increased the activation of BMDCs (60%) relative to TM‐NPs (12%), and the activation ability of BMDCs generated still depended on TLR4 (Figure 3d; Figure S7, Supporting Information), and the activation of TLR4 is not dependent on LPS or bacterial contamination after evaluating the contents of LPS in HM‐NPs. The production of proinflammatory cytokines including IL‐12p70, TNF‐α and IL‐6 increased significantly after BMDCs maturation compared to the control group (Figure 3e–g). Meanwhile, the activation of TLR2 brought by G‐EVLPs and TM‐NPs was significantly reduced after fusion, which may be due to that a certain degree of degradation or loss of nucleic acid fragments occurred o during processing (Figure S7, Supporting Information).

It has been proved that HM‐NPs can promote BMDCs internalization and activation, the ability of HM‐NPs to stimulate an immune response needed to be evaluated. As shown in Figure 3h, we established an assay based on BMDCs and splenic T lymphocytes in vitro. Flow cytometry was used to demonstrate that HM‐NPs can effectively activate T lymphocytes after activating BMDCs, and the number of CD45^+^CD3^+^CD8T^+^ cells increased tens of times significantly relative to the TM‐NPs group (Figure 3i; Figure S8, Supporting Information). The T lymphocytes mentioned above were added to the well plate seeded with tumor cells for 24 h, and Lactate dehydrogenase (LDH) in the supernatant was measured. The results verified that the specific vaccines could specifically produce a targeted effect for killing tumor cells (B16F10 vaccine for B16F10 tumor cells) (Figure 3j–l; Figure S9, Supporting Information). These results indicated that the HM‐NPs can indeed enhance the establishment of adaptive immune responses in vitro.

### 2.3 HM‐NPs Promote DCs Maturation, LNs Accumulation, and Splenic T Cells Activation after Vaccination

**Figure 4:**
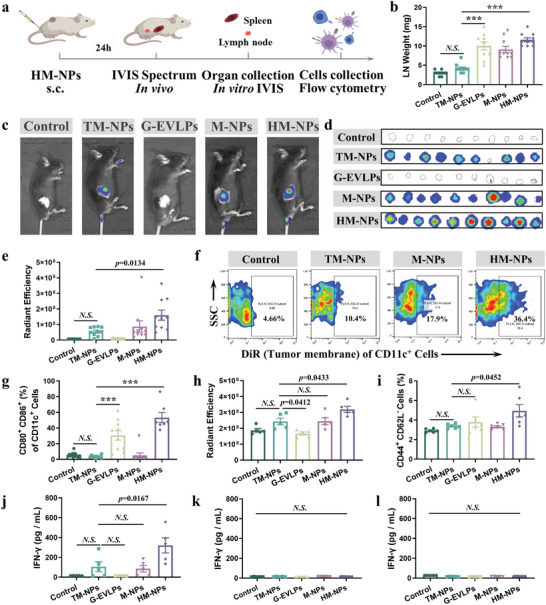
HM‐NPs (B16F10) promote LN accumulation, DCs maturation and splenic T cells activation after vaccination. a) Experimental design to evaluate LN accumulation, DCs maturation and splenic T cells activation. b) Inguinal lymph nodes weight after vaccination (n = 10). c) Fluorescence images after vaccination (DiR‐labeled tumor membrane) (n = 5). d) Fluorescence images of inguinal lymph nodes at after vaccination (DiR‐labeled tumor membrane) (n = 10). e) Fluorescence intensity analysis of inguinal lymph nodes after vaccination (DiR‐labeled tumor membrane). f) Cellular uptake of DiR‐labeled tumor membrane of CD11c^+^ DCs in inguinal lymph nodes, as assessed by flow cytometry (n = 5). g) Flow cytometry analysis of CD11c^+^CD86^+^CD80^+^ DCs in inguinal lymph nodes. h) Fluorescence intensity analysis of spleen after vaccination (DiR‐labeled tumor membrane). i) Flow cytometry analysis quantification of the CD44^hi^CD62L^low^ Tem cells in spleen after vaccination. j–l) IFN‐γ concentration in the supernatant after co‐incubation of the splenic T lymphocytes with B16F10 tumor cells (j), CT26 tumor cells (k) and 4T1 tumor cells (l). Data are representative or pooled and are expressed as Mean ± SE. Asterisks indicate statistically significant differences as analyzed by One‐Way ANOVA (∗∗∗*p* < 0.001, N.S. *p* > 0.05).

Inspired by the results of in vitro experiments, we further conducted experiments in vivo to explore the potential of HM‐NPs for personalized immunotherapy (**Figure** **4**a). We sacrificed the mice and collected inguinal lymph nodes (LNs) 24 h after injecting with various forms of the vaccine at the base of their tails. The results showed that the lymph node mass of the mice in the G‐EVLPs and HM‐NPs group was significantly increased (Figure 4b; Figure S10, Supporting Information). It should be noted that there was no significant change in the spleen weight of the mice (Figure S11, Supporting Information). After using fluorescent dye DiR‐labeled TM‐NPs and using fluorescent dye DiD‐labeled G‐EVLPs, the fluorescent signals were observed that HM‐NPs can significantly increase the targeting of TM‐NPs to inguinal lymph nodes and spleens with IVIS (Figure 4c–e; Figure S12–S13, Supporting Information). After that, we processed the inguinal lymph nodes to obtain single cell suspensions, and founded that the internalization level of HM‐NPs in CD11c^+^ DCs was much higher than that of TM‐NPs by flow cytometry analysis (Figure 4f; Figure S14, Supporting Information). Moreover, the number of mature DCs (CD80 and/or CD86) in inguinal lymph nodes increased significantly after HM‐NPs vaccination (Figure 4g).

We also founded that HM‐NPs preferentially oriented to spleen tissue after vaccination (Figure 4h). As one of the important immune organs, the spleen has the powerful immune memory function.^[^ ^18^ ^]^ Considering that HM‐NPs can promote the maturation and tumor antigens presenting of DCs, the definitive evidence that HM‐NPs vaccines produce effective immune memory remains to be verified. Therefore, we processed the spleens of the vaccinated mice to obtain single cell suspensions, to monitor the changes of T cells during the establishment of adaptive immunity.^[^ ^19^ ^]^ Flow cytometry results showed that HM‐NPs significantly increased the level of Tem (CD44^high^ CD62L^low^) relative to TM‐NPs (Figure 4i). The single cell suspensions mentioned above were added to the well plate seeded with tumor cells for 24 h, and the level of IFN‐γ and LDH in the supernatant was measured. The results proved that the specific vaccines could specifically produce a targeted effect to kill tumor cells (B16F10 vaccine for B16F10 tumor cells) (Figure 4j–l; Figure S15, Supporting Information). That is, HM‐NPs vaccination can indeed promote DCs maturation, LNs accumulation, and splenic T cells activation and establish specific immune memory in vivo.

It should be noted that in the process of building the immune library of the splenic lymphocytes, we found that the mutation frequency and mutation pressure of B cells significantly increased after HM‐NPs vaccination, and the type and number of B cells increased (Figure S16, Supporting Information). The activation of specific anti‐tumor B lymphocytes is more conducive to cytotoxic T lymphocytes to eliminate tumors.^[^ ^20^ ^]^ After vaccination, HM‐NPs could produce tumor‐specific antibodies to label tumor cells and enhance the effectivity of the vaccine. Similar to the previous results of T cell characterization and immune memory T cell activation, the results of immune repertoire also showed that vaccinated with HM‐NPs enhanced the mutation pressure of T cells, and eventually produced more and stronger specific T cells to kill tumors.

### 2.4 HM‐NPs Vaccination Induces Tumor Recurrence Protection in The Murine B16F10 Tumor Model

**Figure 5:**
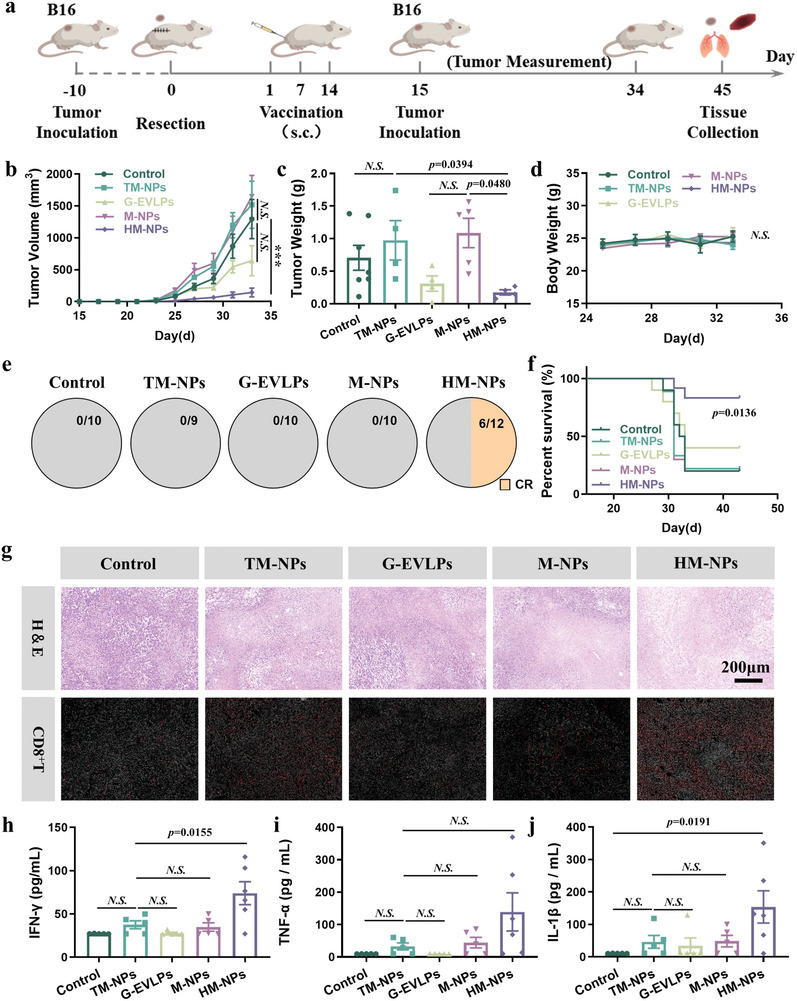
HM‐NPs vaccination induces tumor recurrence suppression in the murine B16F10 tumor model. a) Schematic illustration of the design of the animal experiment. b) Average tumor growth curves of each group in the murine B16F10 tumor model (n = 10). c) Tumor weight of each group in the murine B16F10 tumor model at day 35. d) Body weight of the mice post various treatments (n = 10). e) Tumor complete responses rate of each group (CR: complete responses). f) Survival curves of mice receiving each treatment. g) Photographs are the white filed images of the H&E staining and the immunofluorescence images of CD8^+^ T of the tumors receiving different treatments. h) Proinflammatory IFN‐γ concentration in the serum of the mice receiving each treatment determined by ELISA assay. i) Proinflammatory TNF‐α concentration in the serum of the mice receiving each treatment determined by ELISA assay. j) Proinflammatory IL‐1β concentration in the serum of the mice receiving each treatment determined by ELISA assay. Data are representative or pooled and are expressed as Mean ± SE. Asterisks indicate statistically significant differences as analyzed by One‐Way ANOVA (∗∗∗*p* < 0.001, N.S. *p* > 0.05).

Vaccination with HM‐NPs has been confirmed to activate DCs and T lymphocytes effectively in vivo. Afterward, we need to further evaluate the protection effect of HM‐NPs from tumor recurrence (**Figure** **5**a). The day after three consecutive vaccinations with different vaccine formulations, we rechallenged B16F10 melanoma subcutaneously in mice and continuously measured tumor volumes. As shown in Figure 5b,c,f, the tumor growth of the HM‐NPs group was delayed, exhibiting strong tumor recurrence protection, with the tumor inhibition rate of 86.6% and a survival rate of up to about 83.3% over 45 days (Figure S17, Supporting Information). One thing that needs to be noted is that the mice in the G‐EVLPs group began to die on day 12, and the tumor volume of the dead mice was the largest tumor volume on that day. This resulted in a significant reduction in tumor volume compared with other groups. The body weights of the mice did not change significantly before and after vaccination (Figure 5d). In contrast, tumors of the other vaccine groups showed no growth suppression, and the highest survival rate just reached 40% of the G‐EVLPs group. More importantly, the HM‐NPs vaccine group can significantly increase the complete response rate (CR) of tumors, which reached 50% compared to 0% of the other groups (Figure 5e). Thus, HM‐NPs did exhibit a good ability to protect vaccinated ones from tumor recurrence.

Considering that some tumors of the HM‐NPs group were not completely inhibited, we performed H&E staining and immunohistochemical staining of CD4^+^ and CD8^+^ T lymphocytes on these tumor tissues. The main factor that HM‐NPs significantly delay the tumor growth is the increased infiltration of CD4^+^ and CD8^+^ T lymphocytes into the tumors (Figure 5g and Figure S22a, Supporting Information), and eventually the tumors in HM‐NPs group showed extensive apoptosis and necrosis (Figure 5g) compared to the other groups. Meanwhile, flow cytometry demonstrated that the activation of DCs in inguinal lymph nodes, the levels of memory T_em_ cells in the spleen, and the proportion of CD8^+^ T cells in the tumor were significantly promoted (Figure S19–S22, Supporting Information). In addition, the serum and tumor homogenate concentrations of proinflammatory cytokines, including IFN‐γ, TNF‐α, IL‐6 and IL‐1β, increased significantly in the B16F10 cell–rechallenged mice of the HM‐NPs group (Figure 5h–j; Figure S18, Supporting Information). These results suggested yet a more robust immune memory effect formed, which illustrated the potential of HM‐NPs to combine with other therapies to enhance the vaccine effect.

### 2.5 HM‐NPs Vaccination Inhibits Specific Tumor Recurrence and Provides Long‐Term Anti‐Tumor Protection

**Figure 6:**
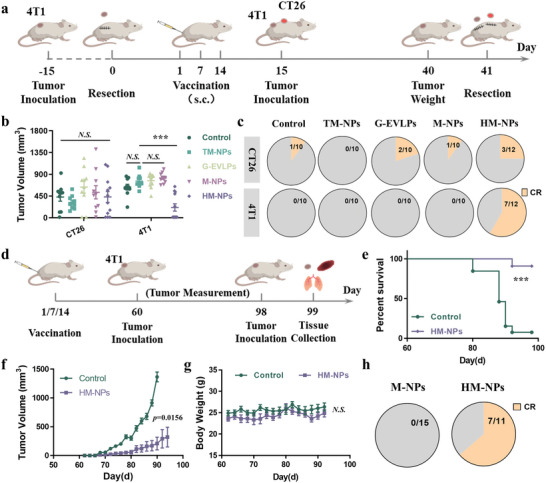
HM‐NPs vaccination inhibits specific tumor recurrence and provides long‐term anti‐tumor protection. a) Schematic illustration of the design of the specific tumor recurrence suppression experiment. b) Tumor volume of CT26 and 4T1 tumors of each group in the murine tumor model. c) Tumor complete responses rate of CT26 and 4T1 tumors of each group (CR: complete responses). d) Schematic illustration of the design of the long‐term anti‐tumor protection experiment. e) Survival curves of mice receiving each treatment. f) Average tumor growth curves of each group in the murine tumor model. g) Body weight of the mice post the two treatments. h) Tumor complete responses rate of 4T1 tumors of each group (CR: complete responses). Data are representative or pooled and are expressed as Mean ± SE. Asterisks indicate statistically significant differences as analyzed by One‐Way ANOVA (∗∗∗*p* < 0.001, N.S. *p* > 0.05).

We further investigated whether HM‐NPs could indeed provide specific tumor recurrence protection in the established bilateral tumor model (**Figure** **6**a). Here we chose the 4T1 breast cancer model with stronger immunosuppression. We vaccinated mice following surgical resection of primary 4T1 tumors, and mice were subsequently inoculated with 4T1 and CT26 tumors simultaneously. HM‐NPs constructed from surgically resected 4T1 tumors only inhibited 4T1 tumor recurrence (Inhibition rate = 69.2%), and the overall complete response rate reached 58.3% (Figure 6b,c; Figure S23, Supporting Information). In contrast, other vaccine formulations have not been shown to suppress tumor growth. More importantly, the immune response and immune memory effect against 4T1 established by HM‐NPs did not inhibit the growth of CT26 tumors (Figure 6b,c; Figure S23, Supporting Information). The complete response rate of each group ranged from 0% to 25%, possibly due to a systemic immune response following vaccination. In other words, HM‐NPs can indeed specifically enhance the vaccine effect of tumor‐associated antigens, which is individual‐specific.

Next, long‐term vaccine protection beyond 60 days has been experimentally investigated (Figure 6d). On day 60 after HM‐NPs vaccination, we re‐inoculated the mice with 4T1 tumor cells, and mice without any treatment were used as the control group. As shown in Figure 6e, HM‐NPs can indeed establish long‐term protection after vaccination to prolong the survival of mice, with an overall survival rate of 90.9%. Meanwhile, the tumor growth was significantly delayed (inhibition rate = 78.6%), the mice in the HM‐NPs group exhibited partial tumor recurrence (36.4%) within 100 days, and the body weights of the mice did not change significantly before and after vaccination (Figure 6f–h; Figure S24, Supporting Information). Therefore, HM‐NPs vaccination establish not only immune response against tumor recurrence, but also a specific long‐term protection.

### 2.6 HM‐NPs Vaccination Inhibits Tumor Metastasis in Murine 4T1 Tumor Models

**Figure 7:**
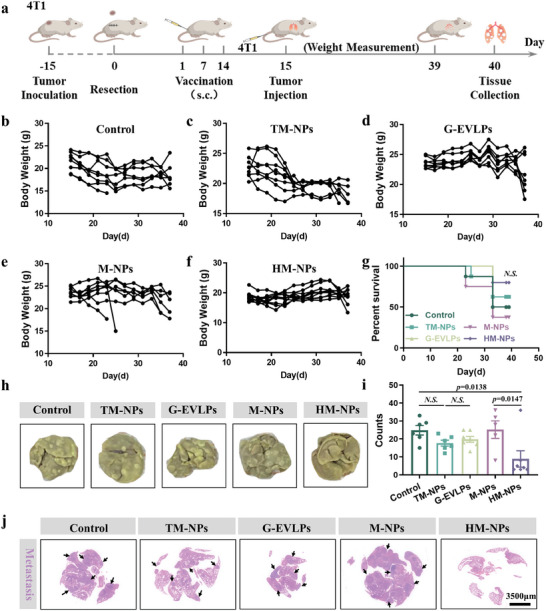
HM‐NPs vaccination inhibits tumor metastasis in murine 4T1 tumor models. a) Schematic illustration of the design of the acute tumor metastasis suppression experiment. b–f) Body weight of each mouse after receiving Saline (b), TM‐NPs (c), G‐EVLPs (d), M‐NPs (e) and HM‐NPs (f). g) Survival curves of mice receiving different treatments. h) Photographs are the white filed images of lung tissues after staining with Bouin's Fluid. i) Quantification of metastatic lesions of the lung tissues. j) Photographs are the panoramic scanning photographs of the H&E staining of lung tissues (Black arrows indicate the metastatic lesions). Data are representative or pooled and are expressed as Mean ± SE. Asterisks indicate statistically significant differences as analyzed by One‐Way ANOVA (N.S. *p* > 0.05).

To demonstrate that the immune memory effect established by HM‐NPs vaccination can effectively reduce the incidence of metastatic tumors, we established the model of acute lung metastasis based on 4T1 tumors (**Figure** **7**a). Primary tumors were removed via surgery at 15 days after inoculation, and the body weight was monitored continuously after vesicles vaccination at the base of the tail and 4T1 cells injection via tail vein. We noticed that the body weights decreased obviously in all other groups except the HM‐NPs group from day 15 to 40, more than half of the mice lost more than 20% of their body weight especially in the TM‐NPs and G‐EVLPs group (Figure 7b–f). This might be the reason that the metastasized tumors affected normal tissues and organs.^[^ ^21^ ^]^ Meanwhile, HM‐NPs vaccination can significantly prolong the survival of lung metastasis model mice to 80% (Figure 7g). As we have imagined, HM‐NPs vaccination could effectively reduce lung metastases, while there are yet a large number of lung metastases in other groups, especially in M‐NPs group (Figure 7h,i; Figure S25, Supporting Information). Panoramic scanning results of H&E sections showed that large areas of metastatic lesions with a condensed nucleus distinct from normal tissues were distributed in the lungs of all groups except Hm‐NPs group (Figure 7j, Supporting Information). Overall, the HM‐NPs vaccination can indeed enhance the anti‐tumor immune response, and can reduce tumor metastases.

### 2.7 HM‐NPs Vaccination Establishes Protection against Orthotopic Mouse MB49 Bladder Tumors

**Figure 8:**
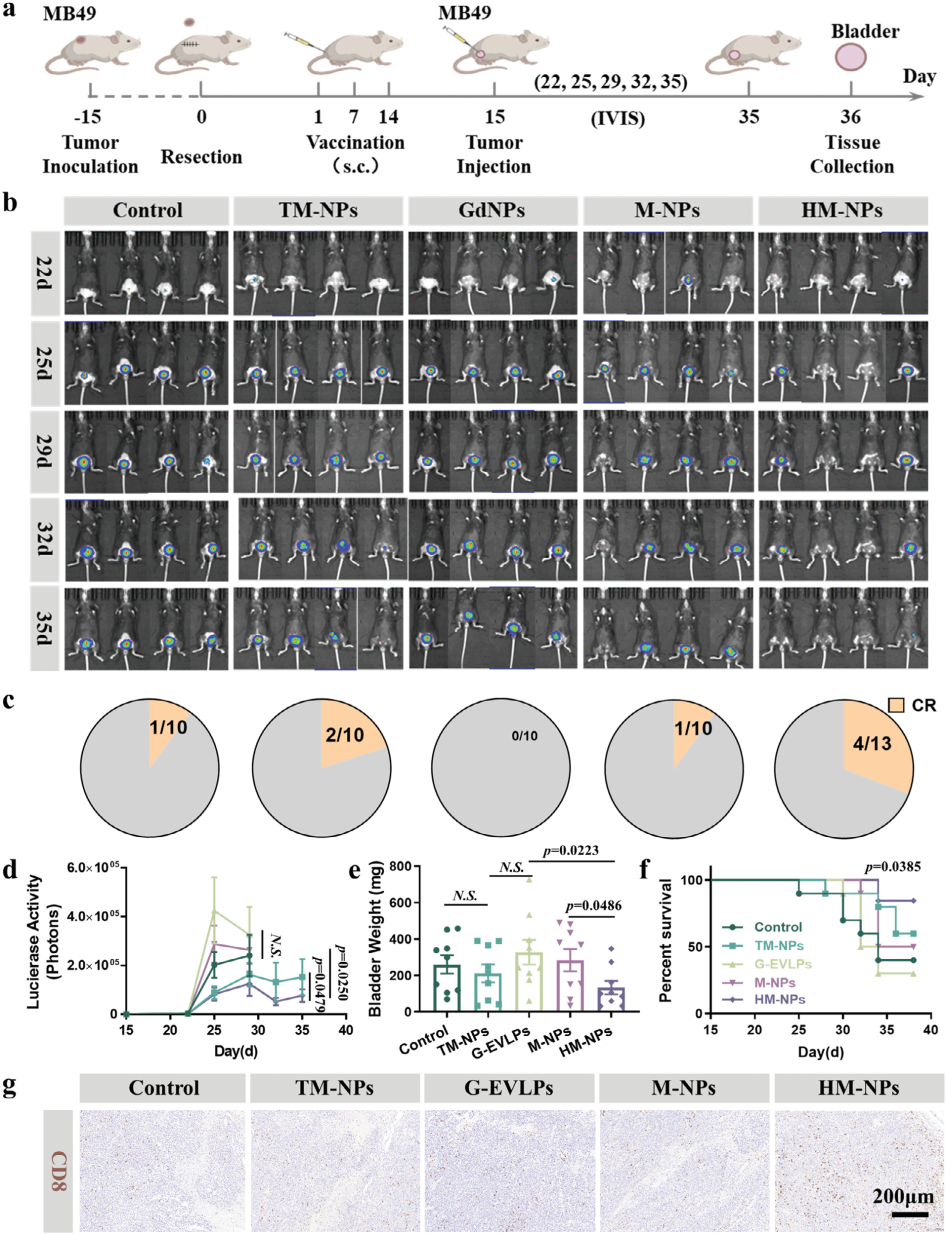
HM‐NPs vaccination establishes protection against orthotopic mouse MB49 bladder tumor. a) Schematic illustration of the design of the orthotopic mouse MB49 1‐Luc bladder tumor experiment. b) IVIS bioluminescence imaging of the mice at days 22, 25, 29, 32, and 35 after vaccination with Saline, TM‐NPs, G‐EVLPs, M‐NPs and HM‐NPs respectively. c) Tumor complete responses rate of the orthotopic mouse MB49 tumors of each group (CR: complete responses). d) Bioluminescence intensity analysis of the orthotopic mouse MB49 tumors at days 22, 25, 29, 32, and 35 after vaccination. e) Tumor weight of each group of the orthotopic mouse MB49 tumors at day 36. f) Survival curves of mice receiving each treatment. g) Immunohistochemical images of the CD8^+^ T cells within tumors post treatments. Data are representative or pooled and are expressed as Mean ± SE. Asterisks indicate statistically significant differences as analyzed by One‐Way ANOVA (N.S. *p* > 0.05).

Subcutaneous tumors do not fully mimic the situation of orthotopic or spontaneous tumors.^[^ ^22^ ^]^ Thus, it is necessary to establish an orthotopic tumor model to clarify the vaccine effect of HM‐NPs. To this end, we transurethral injected female C56BL/6 mice with murine MB49‐Luc bladder tumor cells after the surgical and vaccination procedures described above (**Figure** **8**a). The IVIS images showed that the tumor volumes gradually increased in all groups except for HM‐NPs vaccination groups (Figure 8b,d; Figure S26–S30, Supporting Information). Meanwhile, the HM‐NPs vaccination significantly increased the complete response rate to 33.3%, rather than 20% of TM‐NPs vaccination (Figure 8c). We found that the weight of the bladders in the HM‐NPs group was significantly lower than that in the other groups after sacrificing the mice to collect the bladders. (Figure 8e; Figure S31a, Supporting Information). The H&E staining images and TUNEL fluorescence images of the bladder obtained above showed that HM‐NPs yet had the certain protective effect, because there were still a large number of CD4^+^ and CD8^+^ T lymphocytes infiltrating (Figure 8g; Figure S31b, Supporting Information). Combining with other therapies may further stimulate the potential of HM‐NPs vaccines.^[^ ^23^ ^]^ As shown in Figure S32 (Supporting Information), Bladder cancer increased the burden on mice during growth and caused weight loss in mice, which excluded the HM‐NPs vaccine group. On the whole, the above results confirmed that HM‐NPs can not only play a protective role in subcutaneous tumors and metastatic tumors, but also play an effective protective role in orthotopic tumors, and ultimately prolong survival rate.

### 2.8 HM‐NPs Vaccination Exhibits A Favorable Safety Profile

**Figure 9:**
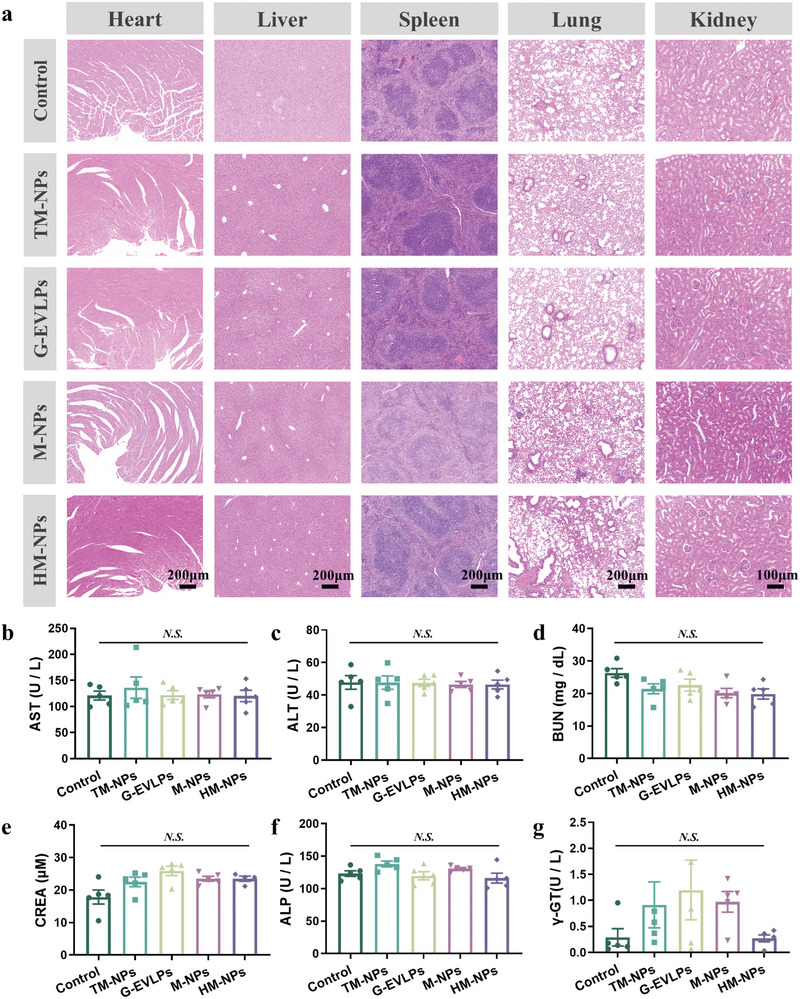
HM‐NPs vaccination exhibits a favorable safety profile. a) Photographs of the H&E staining of different organs of the tumor‐bearing mice receiving each treatment. b) Level of blood aspartate aminotransferase (AST) of the mice receiving each treatment at day 7. c) Level of blood alanine aminotransferase (ALT) of the mice receiving each treatment at day 7. d) Level of blood urea nitrogen (BUN) of the mice receiving each treatment at day 7. e) Level of blood creatinine (CREA) of the mice receiving each treatment at day 7. f) Level of blood alkaline phosphatase (ALP) of the mice receiving each treatment at day 7. g) Level of blood γ‐glutamyl transpeptidase (γ‐GT) of the mice receiving each treatment at day 7. Data are representative or pooled and are expressed as Mean ± SE. Asterisks indicate statistically significant differences as analyzed by One‐Way ANOVA (N.S. *p* > 0.05).

Considering that G‐EVLPs are vesicle‐like substances derived from ginseng, we need to verify the safety of the prepared HM‐NPs in mammals. Although there was no significant high level of systemic inflammation, we further evaluated the in vivo safety of G‐EVLPs and HM‐NPs. First, the HM‐NPs we prepared did not induce hemolysis at the concentration we used. Second, after vaccinating HM‐NPs, there were no significant changes in the main organs (heart, liver, spleen, lung and kidney) of the mice (**Figure** **9**a). Thirdly, as shown in Figure 9b–g, all of the biochemical blood indicators were not significantly different from the normal values, which demonstrated that no significant heart, liver and kidney damage occurred following vaccination. These findings highlight the pronounced safety advantage of HM‐NPs vaccination, which may be clinically valuable.^[^ ^1^ ^]^

## 3 Conclusion

The emergence of tumor vaccines provides a new way for tumor immunotherapy, and also provides inhibitory strategies for primary tumors, tumor recurrence and metastasis.^[^ ^24^ ^]^ Here, we fused ginseng‐derived extracellular vesicles‐like particles (G‐EVLPs) with surgically derived tumor cell membranes (TM‐NPs), and obtained the HM‐NPs with properties of both G‐EVLPs and TM‐NPs. HM‐NPs are spherical particles with a vesicle‐like structure. The obtained HM‐NPs are a formulation that simultaneously deliver tumor‐associated antigens and immune adjuvants to DCs, and ultimately unintentionally achieving the goal of utilizing plants for cross‐species immune regulation. Compared with somatic cell‐derived vesicles and/or blood cell‐derived vesicles, plants‐derived vesicles are more heterogeneous to be internalized by DCs and activate DCs to promote their maturation.^[^ ^25^ ^]^ Compared with microbial‐derived vesicles, plants‐derived nanovesicles have better biocompatibility because ginseng has the medicinal and food homology.^[^ ^26^ ^]^ That is to say, plants‐derived nanovesicles have the superiority when composing the fusion vaccine

As a fusion tumor vaccine, it is also necessary to pay attention to the potential biocompatibility of HM‐NPs. Our previous studies have shown that G‐EVLPs have the ability to activate monocytes through TLR4, and the TLR4‐activating was not due to LPS as demonstrated.^[^ ^11^ ^]^ This hybrid vesicle still maintains the activation activity on TLR4, which illustrates the potential of G‐EVLPs as drug carrier with adjuvant properties. More importantly, the average particle size of G‐EVLPs is 165 nm after optimizing the preparation process, and the average particle size of the hybrid vaccine HM‐NPs is only 200 nm. This also means that sterile filtration can be performed by using a 220 nm filter to obtain an injectable formulation of HM‐NPs.^[^ ^27^ ^]^ At the same time, during the peri‐injection period of HM‐NPs, the body weight of the mice did not change significantly, nor did it induce a strong cytokine storm, nor did it induce damage to major tissues and organs. Therefore, the fusion membrane vaccine HM‐NPs had good biocompatibility.

Nowadays, surgical resection combined with radiotherapy and/or chemotherapy is still the first choice for most patients with solid tumors clinically. It has been reported that through the screening of tumor neoantigens or tumor‐associated antigens, fusion proteins or mRNA vaccines with strong therapeutic effects can be finally established.^[^ ^28^ ^]^ This process yet requires the establishment of a co‐delivery system for tumor antigens and adjuvants. It is worth noting that the screening process for neoantigens or tumor‐associated antigens is time‐consuming, costly, and has a high clinical entry threshold.^[^ ^29^ ^]^ The prospect of clinical translation in a short term is relatively weak. Nevertheless, the surgically obtained tumor membrane will directly retain tumor‐associated antigens and tumor neoantigens simultaneously, since the surgical resection method is commonly used in clinical practice.^[^ ^30^ ^]^ Using a simple method and relatively cheap raw plants‐derived G‐EVLPs, the hybrid vaccine HM‐NPs has relatively favorable clinical translation potential.

The overall design still remains expanses for advanced optimization. First, the immunosuppressive microenvironment within the growing tumor was not taken into account.^[^ ^31^ ^]^ Although HM‐NPs do have a relatively strong protective ability against tumor recurrence and tumor metastasis. The tumor volume of some mice was not completely suppressed, even though an increased recruitment of intratumoral T lymphocytes formed. Therefore, combining with conventional immunotherapy, such as PD‐L1 and/or other immune checkpoint blockers for synergistic treatment is a desirable option. Second, tumor metastasis models with urgent clinical needs can be selected.^[^ ^32^ ^]^ For instance, 70% of lung cancer patients will have leptomeningeal metastasis with short overall survival,^[^ ^33^ ^]^ and the lifetime after conventional therapies might be guaranteed by combining the HM‐NPs vaccination. Thirdly, xenograft tumor models could be established to prove the effectiveness of HM‐NPs in human tumors to enable the clinical transformation potential.

In conclusion, we prepared a hybrid vaccine HM‐NPs for personalized immune therapy by fusing ginseng‐derived extracellular vesicles‐like particles (G‐EVLPs) with surgically derived tumor cell membranes (TM‐NPs). The obtained HM‐NPs are co‐delivery formulations of adjuvant from G‐EVLPs and antigens from TM‐NPs. The adaptive immune response can be significantly stimulated, and specific cytotoxic T lymphocytes can be produced to kill tumor cells after inoculation of HM‐NPs vaccine. Vaccination with HM‐NPs generated stronger immune memory and provided long‐term protection against specific tumors, and protected the vaccinated ones from tumor recurrence or tumor metastasis. Moreover, HM‐NPs have good biocompatibility and are convenient to be prepared clinically. Thus, the personalized hybrid HM‐NPs vaccine with natural adjuvant properties offers an opportunity for the development of individualized cancer vaccines for a broad range of solid tumors in clinic someday.

((References should be superscripted and appear after punctuation.^[^ ^1^, ^2^ ^]^ If you have used reference management software such as EndNote to prepare your manuscript, please convert the fields to plain text by selecting all text with [ctrl]+[A], then [ctrl]+[shift]+[F9]).^[^ ^3^, ^4^, ^5^ ^]^ Footnotes should not be used in the text. Instead, additional information can be added to the Reference list. Please define all acronyms except IR, UV, NMR, and DNA or similar (for a list of acronyms not requiring definition, please see the list available on the journal homepage in our “Author Guidelines” section.))

## 4 Experimental Section

### Materials

Reagents: Fresh ginseng roots were purchased from Panax ginseng Base (Wanshan, Jilin, China). Anti‐CD11c antibody, anti‐CD86 antibody, anti‐CD80 antibody, anti‐CD45 antibody, anti‐CD3 antibody, anti‐CD4 antibody, anti‐CD8 antibody, anti‐CD11b antibody, anti‐F4/80 antibody, anti‐CD44 antibody and anti‐CD62L antibody were purchased from Biolegend. Roswell Park Memorial Institute 1640 (RPMI 1640), fetal bovine serum (FBS), Trypsin‐EDTA and penicillin were purchased from Gibco (Thermo fisher, US). Dimethyl sulfoxide (DMSO) and ethanol were obtained from Sinopharm Chemical Reagent Co. Anti‐TLR2 and anti‐TLR4 antibody, anti‐β‐actin antibody provided by Abcam Corporation. IL‐12p70 ELISA Kit, TNF‐α ELISA Kit, IL‐6 ELISA Kit, IL‐1β ELISA Kit, IFN‐γ ELISA Kit was purchased from Biolegend. DiO, DiI, DiR, DAPI were supplied by KeyGEN BioTECH. LDH Assay Kit, SDS‐PAGE Gel Kit, Protein Gel Flash Staining Kit, and Micro NO Content Assay Kit were supplied by Keygen BioTech. All other reagents were obtained from Sigma‐Aldrich.

Cell line and Cell Culture: B16F10 cells, 4T1 cells, and CT26 cells were obtained from the Cell Bank of Shanghai Institutes for Biological Sciences, Chinese Academy of Sciences (Shanghai, China). Luciferase MB49 was donated by Nanjing University. 4T1 cells were cultured in DMEM medium, B16F10, MB49 and CT26 cells were cultured in RPMI 1640 medium supplemented with 10% fetal bovine serum and 1% L‐glutamine (200 mM). Passaging the B16F10, CT26, MB49 and 4T1 cells when the confluence of the cells came to 60–80%.

Animals: Balb/c and C57BL/6 mice were purchased from GemPharmatech (Nanjing, China) and Yangzhou University (Yangzhou, China), the weight of the mice was ≈ 18–22 g. All animal tests and experimental procedures were conducted according to the Administration Committee of Experimental Animals in Jiangsu Province and the Ethics Committee of Nanjing University of Chinese Medicine (AEWC‐20220329‐198).

### Purification of G‐EVLPs

For isolation of G‐EVLPs, fresh ginseng roots were purchased from Panax ginseng Base (Wanshan, Jilin, China) and washed with deionized water for three times at RT. After the final wash, ginseng roots were squeezed in a low‐speed breaker to obtain ginseng fluid. Then, the obtained ginseng fluid was sequentially centrifuged at 200×g for 10 min, 2000×g for 20 min and 10000×g for 30 min (three times) to remove large particles and fibers. The final supernatant was ultracentrifuged at 100000×g for 60 min (Beckman Optima XE‐100, Beckman, USA), and the pellets were resuspended in PBS (5:1), which were then transferred to a gradient sucrose solution (15, 30, 45, and 60%) and ultracentrifuged at 150000×g for another 60 min. The band at the 45% sucrose layer was collected and defined as G‐EVLPs according to TEM (transmission electron microscopy) examination. The resuspension was filtered (0.22 µm microporous membrane) and stored at −80 °C. Quantification of G‐EVLPs using the BCA kit.

### Initial Tumor Models Establishment and TM‐NPs Extraction

C57BL6 and Balb/c mice were raised with free access to abundant food and water. To establish B16F10, CT26, 4T1 and MB49 tumor models, fur of the Balb/c and C57BL6 mice was carefully removed. 5×10^5^ B16F10, CT26, 4T1, and MB49 tumor cells of 0.05 ml were then injected subcutaneously into the oxter flank of the mouse. When the tumor volume reached ≈ 100–150 mm^3^, the surgeries were conducted on these mice. The tumor after surgical resection was cut into small pieces, and then digested with collagenase II, DNase and neutral protease to obtain the single cell suspension. The cell suspension obtained was passed through a 70 µm filter to obtain uniformly dispersed cells.

For isolation of TM‐NPs, the obtained cells were resuspended in PBS supplemented with DNase, and were disrupted using a ultrasonic cell disruptor in an ice bath (150 W, 3 min). Then, the obtained solution was sequentially centrifuged at 2000×g for 20 min and 10000×g for 30 min to remove large particles. The final supernatant was ultracentrifuged at 100000×g for 60 min (Beckman Optima XE‐100, Beckman, USA), and the pellets were resuspended in PBS, which were then transferred to a gradient sucrose solution (15, 30, 45, and 60%) and ultracentrifuged at 150000×g for another 60 min. TM‐NPs were obtained, the TM‐NPs solution was filtered by a 0.22 µm microporous membrane. Quantification of TM‐NPs using the BCA kit.

### Synthesis and Characterization of HM‐NPs

TM‐NPs and G‐EVLPs were dispersed in 1×PBS with a mass ratio (by protein) of 5:1 (5:3, 5:5). Then, the mixed solutions were physically extruded for 11 times using the extruder under 20°C by an Avanti mini extruder through a porous polycarbonate membrane (200 nm and 400 nm). The mixture was concentrated using a freeze concentrator to obtain the HM‐NPs. By dynamic light scattering (DLS, 90Plus, Brookhaven Instrum. Corp), the surface potential (ζ potential) and the particle sizes of nanoparticles were measured. The structures of the sample were characterized by TEM (Tecan G2 F20 S‐Twin). Membrane Fusion Characterization Analysis by SDS‐PAGE Electrophoresis and through fluorescence resonance energy transfer (FRET) technology.

### In Vitro BMDCs Uptake and Maturation Experiment

BMDCs were generated from bone mesenchymal stem cells harvested from C57BL/6 mice. The process was consistent with the previous protocol described.^[^ ^34^ ^]^ BMDCs were pre‐seeded in 6‐well plates at a density of 1×10^5^ per well and incubated for 24 h. DiD‐labeled tumor membrane were prepared for the following process. A certain amount of TM‐NPs (50 µg ml^−1^) and G‐EVLPs (10 µg ml^−1^) were incubated with BMDCs for 24 h.

The uptake of DID‐labeled TM‐NPs by BMDCs was observed by confocal laser microscopy and was checked by Flow Cytometry. After incubating with HM‐NPs, the cells were then collected by centrifugation and resuspended in FACS buffer. CD11c, CD80, and CD86 were used for fluorescence labeling and analyzed using a BD FACS Aria II flow cytometer. The cytokines in the supernatant of the BMDCs were determined by ELISA kit.

### In Vivo DCs Uptake and Maturation Experiment

The B16F10 melanoma model was formed and tumor membrane was collected as above mentioned. TM‐NPs and G‐EVLPs were dispersed in 1×PBS with a mass ratio (by protein) of 5:1, the dosage of G‐EVLPs were 100 µg (by protein). The concentration of G‐EVLPs used subsequently will be this concentration as the final concentration (0.1 mL per mice). DiD‐labeled tumor membrane was prepared for the following process. The mice were immunized with the different vaccine formulations and targeting delivery of tumor membrane to lymph nodes and spleen was determined by near infrared imaging. Lymph nodes were surgically removed to prepare single‐cell suspensions, and flow cytometry was used to analyze CD11c^+^CD80^+^CD86^+^ mature DCs. Spleens were surgically removed to prepare single‐cell suspensions, and flow cytometry was used to analyze CD45^+^ CD3^+^CD8^+^ CD44^high^CD62L^low^ T cells

### T Cells Response Measurements

BMDCs were generated from bone mesenchymal stem cells harvested from C57BL/6 mice and were pre‐seeded in 6‐well plates at a density of 1×10^5^ per well and incubated for 24 h. The different vaccine formulations were incubated with BMDCs for 24 h. Spleens were surgically removed to prepare single‐cell suspensions, which were then added into the plates above. After incubation for 24 h, the proportion of CD45^+^CD3^+^CD8^+^ T cells was determined by flow cytometry. The upper layer of the medium was added to the plates pre‐seeded with tumor cells (3×10^4^ per well in 24‐well plates), and the concentration of IFN‐γ and LDH in the supernatant were detected by ELISA and Kit, respectively.

The B16F10 melanoma model was formed and tumor membrane was collected as above mentioned. After injecting with different vaccine formulations into the base of the tail, the spleens of the mice were collected to prepare single‐cell suspensions after 24 h, which were added to the plates pre‐seeded with tumor cells (3×10^4^ per well) for another 24 h. The concentration of IFN‐γ and LDH in the supernatant were detected by ELISA and Kit, respectively.

### In Vivo Evaluation of Tumor Inhibition

*Primary tumor models*: Tumor‐bearing mice were subcutaneously inoculated with B16F10, 4T1 and MB49 tumor (3×10^5^ cells per mouse) to obtain primary tumor models as above.

### Tumor Recurrence Regression Experiments (B16F10)

The B16F10 melanoma cancer model was formed and tumor membrane was collected at day 10 as above mentioned. Different vaccine formulations were prepared following the process above and were injected into the base of the tail for vaccination. At day 15, the mice were inoculated with B16F10 melanoma cancer ((3×10^5^ cells per mouse, n = 10 mice per group). The volumes of all tumors and the weight of mice were measured with electronic calipers and scale every other day. Tumor volume was calculated according to the following formula: V = (length×width×width)/2. H&E staining, immunohistochemistry of CD4^+^ T cells and CD8^+^ T cells in the resected mouse tumors was detected at 35 days.

### Bilateral Tumor Experiment (4T1 and CT26)

The 4T1 breast cancer model was formed and tumor membrane was collected at day 15 as above mentioned. Different vaccine formulations were prepared following the process above and were injected into the base of the tail for vaccination. At day 15, the mice were inoculated with 4T1 tumor and CT26 tumor (3×10^5^ cells per mouse, n = 10 mice per group). The tumor weight of mice was measured with electronic scale at day 40. The two tumors of each mouse were resected at day 41 and a new 4T1 tumor was inoculated (3×10^5^ cells per mouse). The volumes of all tumors and the weight of mice were measured with electronic calipers and scale every other day. Tumor volume was calculated according to the following formula: V = (length×width×width)/2.

### Metastatic Tumor Model (Luc‐4T1)

The primary 4T1 breast cancer model and different vaccine formulations were prepared following the process above. At day 15, the mice were intravenously injected with 1×10^5^ Luc‐4T1 tumor cells through tail vein. An IVIS (PerkinElmer) was used to monitor the bioluminescence signal generated by the 4T1‐Luc cells and the body weights were measured with electronic scale every other day. After the mice were sacrificed on the 40th day, the lung and liver tissues of the mice were collected. Lungs and livers were stained using Bouin's fluid and the number of metastases were counted. And H&E staining of lung tissues.

### Orthotopic Tumor Recurrence Regression Experiments (MB49)

The primary MB49 bladder cancer model and different vaccine formulations were prepared following the process above. At day 15 after vaccination for 3 times, the mice were transurethral injected with 1×10^5^ Luc‐MB49 tumor cells. An IVIS (PerkinElmer) was used to monitor the bioluminescence signal generated by the Luc‐MB49 cells and the body weights were measured with electronic scale every other day. After the mice were sacrificed on the 36th day, the tumor tissues of the mice were collected and weighted. Immunohistochemistry of CD4^+^ T cells and CD8^+^ T cells in the resected mouse bladders was detected.

### Serum Cytokines Measurements and Blood Index Detection

The B16F10 melanoma was inoculated into female C57BL6 mice and tumor membrane was collected as above mentioned to obtain different vaccine formulations. After injecting with different vaccine formulations, the concentrations of inflammatory cytokines and chemokines in the serum were detected using ELISA kit (IFN‐γ, TNF‐α, IL‐6 and IL‐1β). The concentrations were normalized to total protein quantity.

### Data Availability and Statistical Analysis

The authors declare that the main data supporting the findings of this study were available within the article and its Supplementary Information. Additional data were available from the corresponding author upon request. Student's t test was used for comparison between two groups. One‐way analysis of variance (ANOVA) was used for multiple comparisons. Survival curves were assessed with a log‐rank (Mantel‐Cox) test. Statistical significance was set as follows: * *p* < 0.05, ** *p* < 0.01, *** *p* < 0.001, and N.S. denotes no significant difference.

## Conflict of Interest

The authors declare no conflict of interest.

## Supporting information

Supporting Information

## Data Availability

The data that support the findings of this study are available from the corresponding author upon reasonable request.
