# Supplementary material for: Hybrid Ginseng‐derived Extracellular Vesicles‐Like Particles with Autologous Tumor Cell Membrane for Personalized Vaccination to Inhibit Tumor Recurrence and Metastasis
Source: Adv Sci (Weinh). 2024 Feb 14;11(17):2308235. doi: 10.1002/advs.202308235 (PMC11077655; doi:10.1002/advs.202308235)
Supplement: Supplementary file 1 — Supporting Information [file ADVS-11-2308235-s001.pdf]

ADVANCED SCIENCE
Open Access

Supporting Information

for *Adv. Sci.*, DOI 10.1002/advs.202308235

Hybrid Ginseng-derived Extracellular Vesicles-Like Particles with Autologous Tumor Cell Membrane for Personalized Vaccination to Inhibit Tumor Recurrence and Metastasis

Haoran Wang, Jiankang Mu, Yexing Chen, Yali Liu, Xianghui Li, Hao Li and Peng Cao**

Supporting Information

Hybrid Ginseng-derived Extracellular Vesicles-like Particles with Autologous Tumor Cell Membrane for Personalized Vaccination to Inhibit Tumor Recurrence and Metastasis

*Haoran Wang**, *Jiankang Mu, Yexing Chen, Yali Liu, Xianghui Li, Hao Li, Peng Cao**

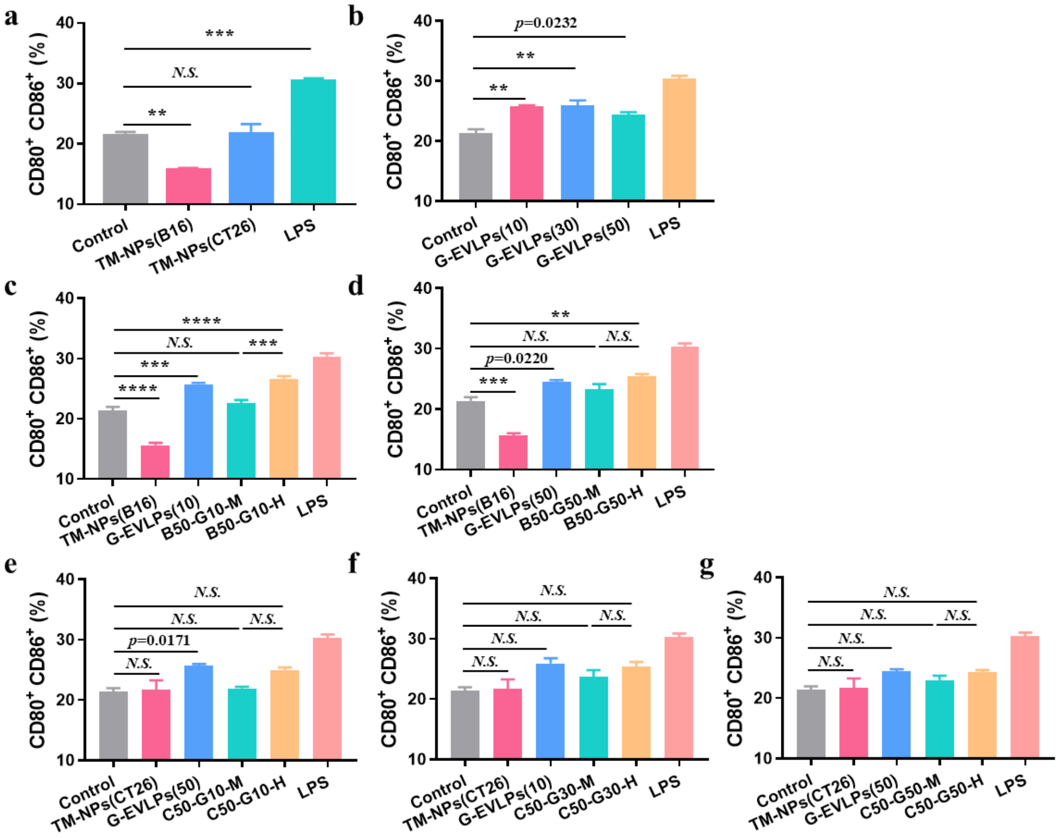

Figure S1. Flow cytometry analysis of BMDCs activation by G-EVLPs, TM-NPs and HM-NPs. The specific numbers in the figure represent the final concentration of G-EVLPs (10: 10 μg/mL, 30: 30 μg/mL and 50: 50 μg/mL). M stands for the simply mixed NPs (M-NPs). H stands for the fused HM-NPs. The final concentration of LPS is 50 μg/mL. Data are representative or pooled and are expressed as Mean ± SE. Asterisks indicate statistically significant differences as analyzed by One-Way ANOVA (**** *p* and *** *p* < 0.001, ** *p* < 0.01, N.S. p>0.05)

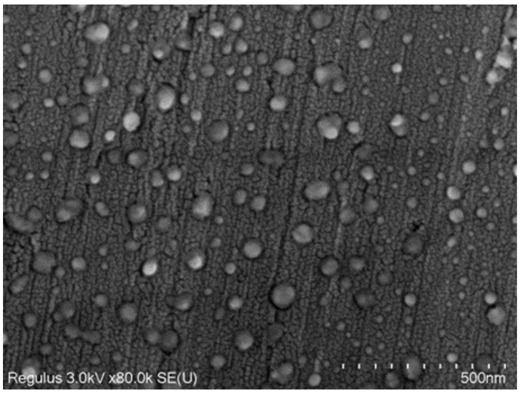

Figure S2. Frozen scanning electron micrograph of HM-NPs.

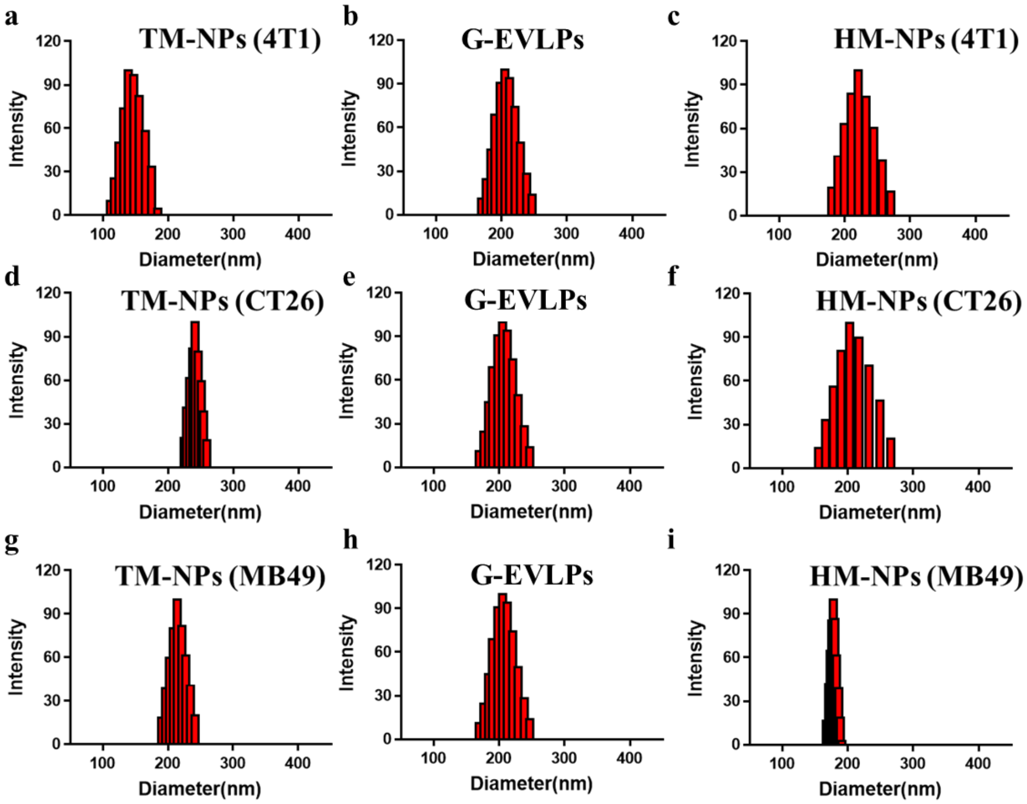

Figure S3. Particle size distribution of TM-NPs, G-EVLPs and HM-NPs determined by DLS. a. Particle size distribution of TM-NPs (derived from 4T1 cells). **b.** Particle size distribution of G-EVLPs. **c.** Particle size distribution of HM-NPs (derived from 4T1 cells). **d.** Particle size distribution of TM-NPs (derived from CT26 cells). **e.** Particle size distribution of G-EVLPs. **f.** Particle size distribution of HM-NPs (derived from CT26 cells). **g.** Particle size distribution of TM-NPs (derived from MB49 cells). **h.** Particle size distribution of G-EVLPs. **i.** Particle size distribution of HM-NPs (derived from MB49 cells).

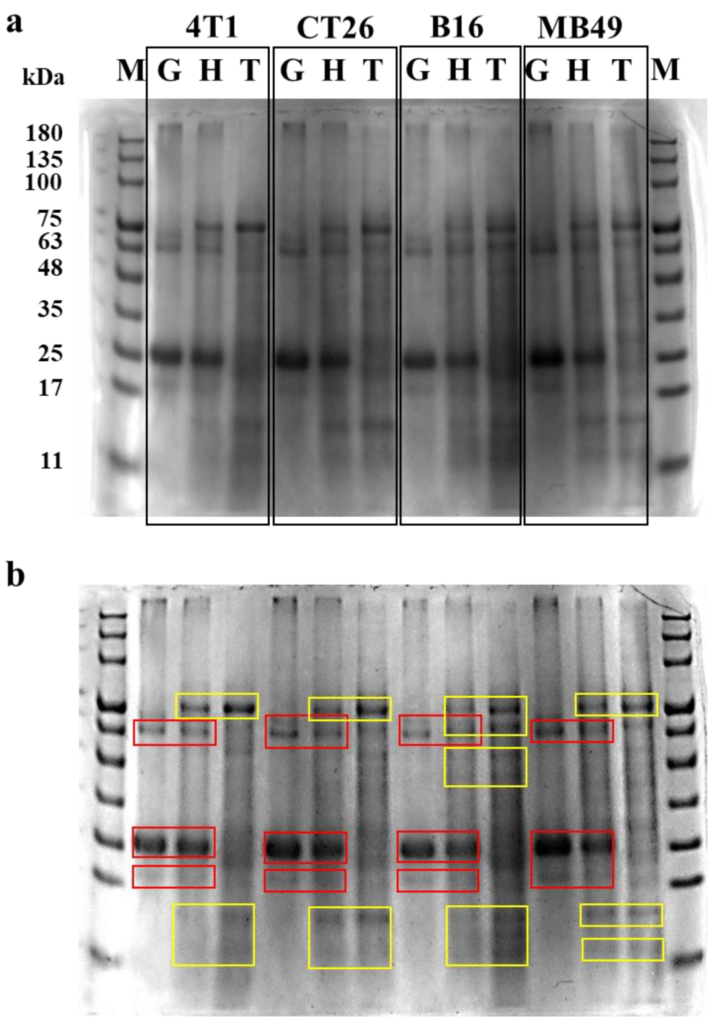

Figure S4. SDS-PAGE protein analysis of Marker (M), G-EVLPs (G), TM-NPs (T) and HM-NPs (H). **a.** Images of SDS-PAGE protein analysis. b. Images of SDS-PAGE protein analysis marked for the same protein (The red box indicates the same G-EVLPs protein band, while the yellow box indicates the same tumor cell membrane protein band).

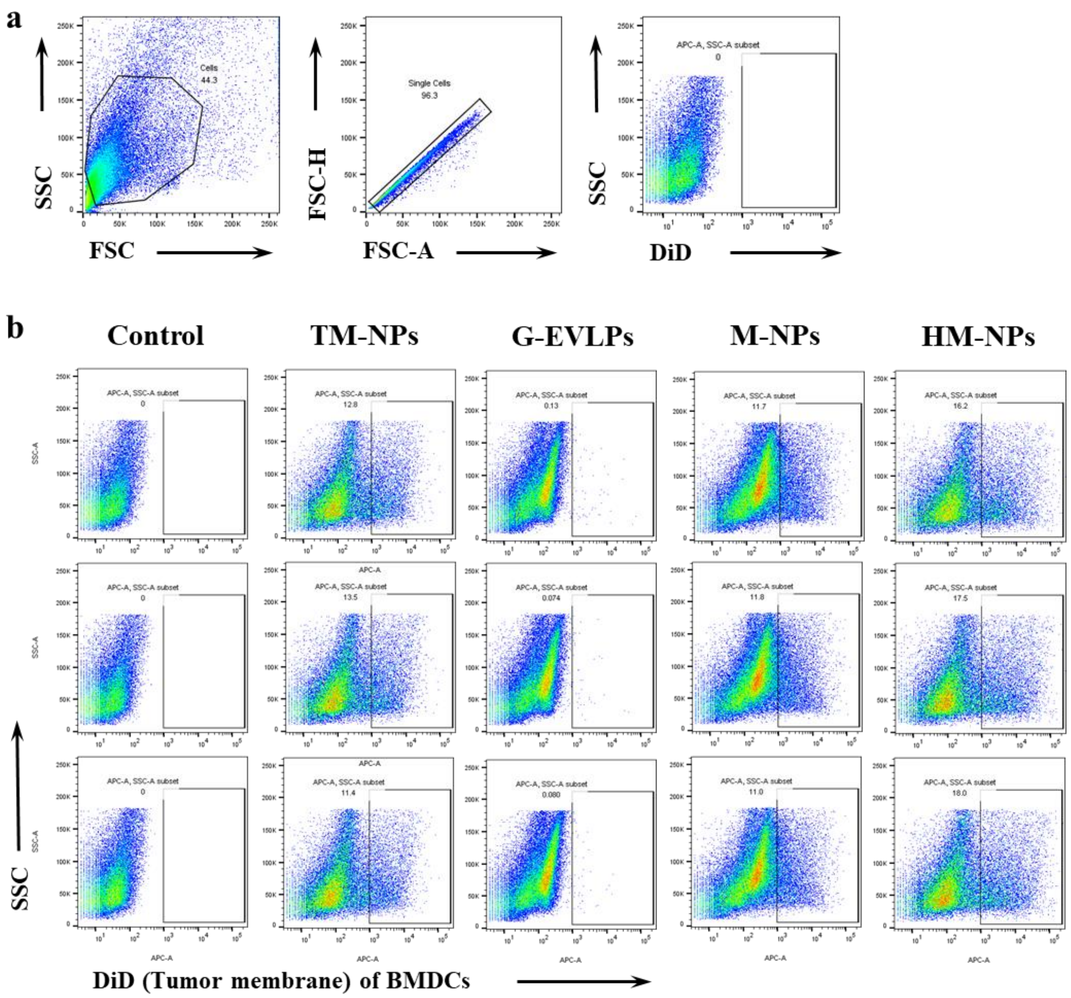

Figure S5. Flow cytometry analysis of cellular uptake of DiD-labeled tumor membrane after incubation with different NPs for 24 hours. a. Principles of flow cytometry data processing. **b.** Flow cytometry uptake data for different groups.

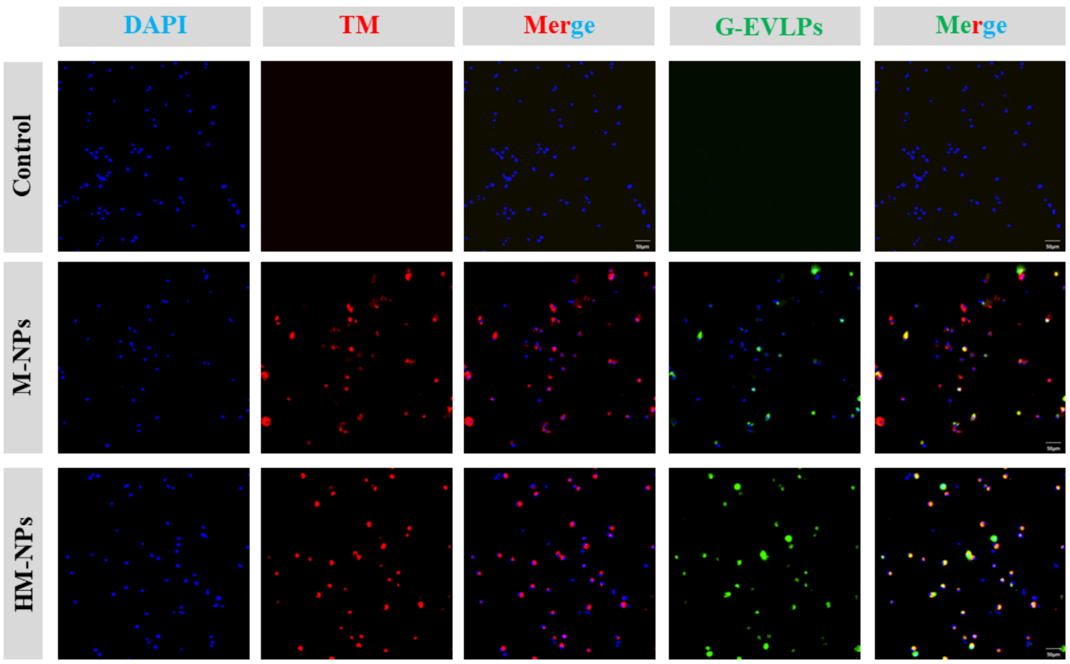

Figure S6. Colocalization analysis of M-NPs and HM-NPs after a 24-hour incubation (DiD-labeled tumor membrane).

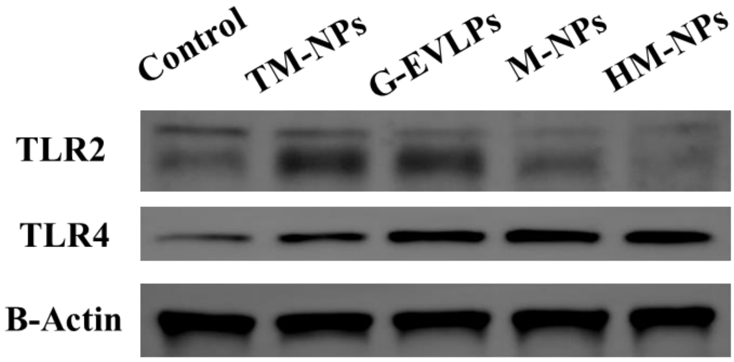

Figure S7. Western blot analysis of TLR2 and TLR4 proteins of BMDCs receiving different process. The gels were loaded with equal amounts of the proteins (10 μg).

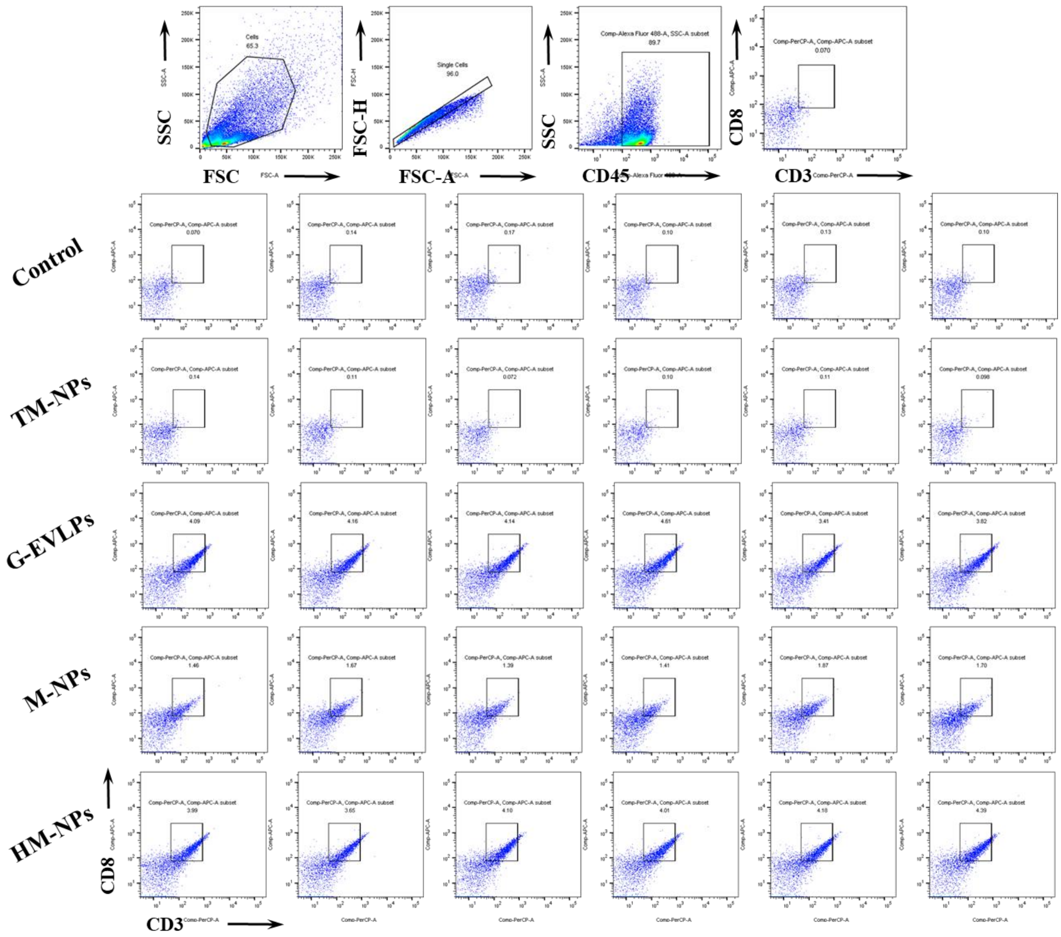

Figure S8. Flow cytometry analysis of CD45$^+$CD3$^+$CD8$^+$ T cells of the specific immune activation experiments *in vitro*.

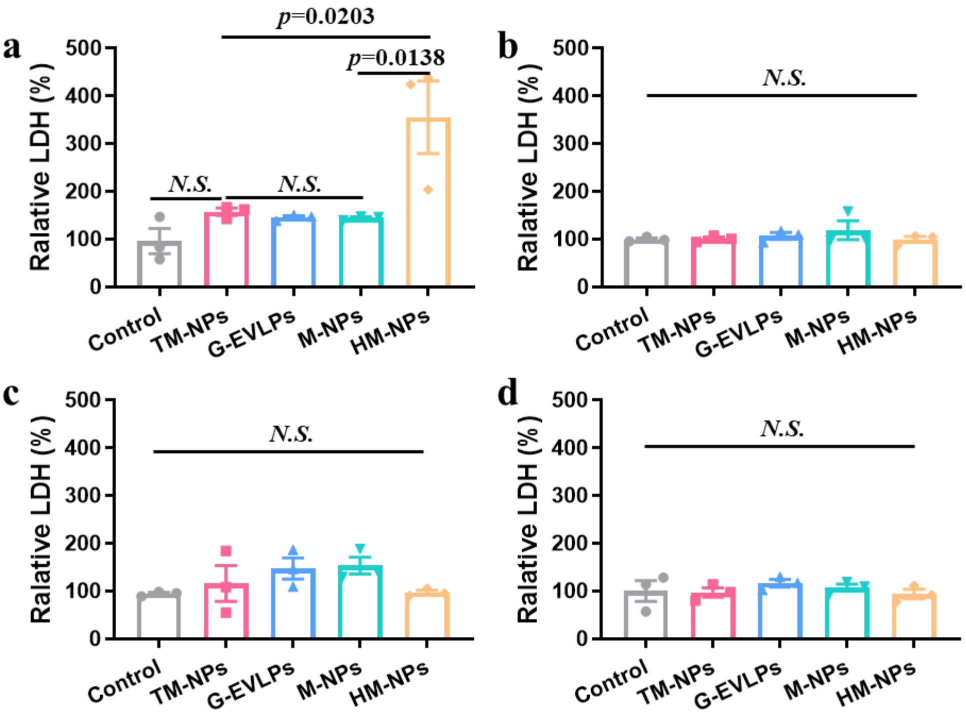

Figure S9. Specific immune activation determined by LDH kit. LDH concentration in the supernatant after co-incubation of the splenic T lymphocytes with B16F10 tumor cells (a), CT26 tumor cells (b), MB49 tumor cells (c) and 4T1 tumor cells (d). Data are representative or pooled and are expressed as Mean ± SE. Asterisks indicate statistically significant differences as analyzed by One-Way ANOVA (N.S. p>0.05).

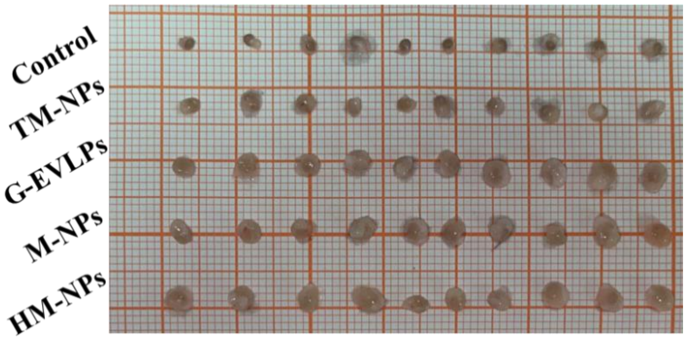

Figure S10. Photographs of the inguinal lymph nodes after vaccination after 24 h.

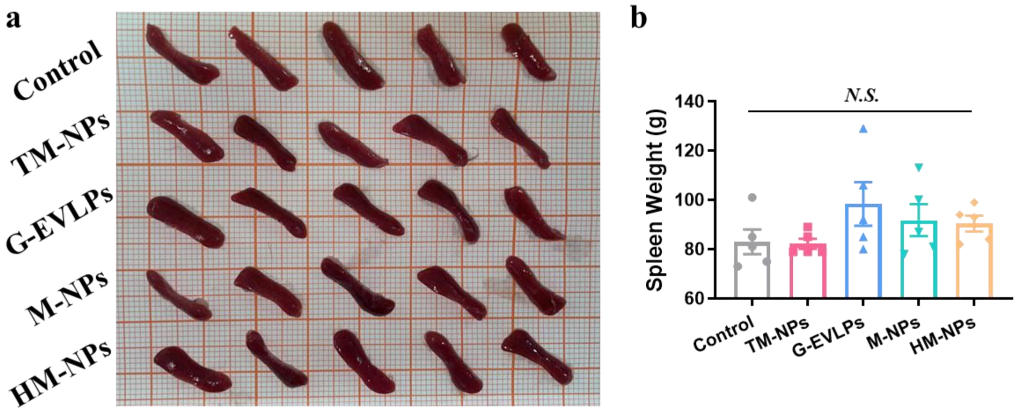

Figure S11. Photographs of the spleens and the spleens weight after vaccination after 24 h. Data are representative or pooled and are expressed as Mean ± SE. Asterisks indicate statistically significant differences as analyzed by One-Way ANOVA (N.S. $p>0.05$).

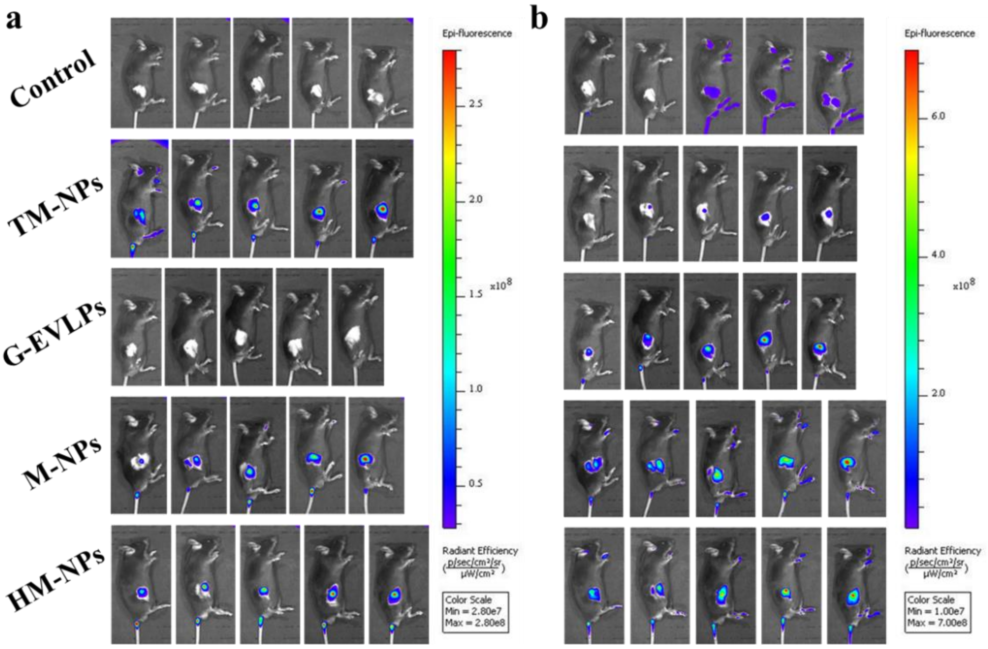

Figure S12. Distribution of different NPs in lymph nodes. a. Fluorescence images after vaccination (DiR-labeled tumor membrane). b. Fluorescence images after vaccination (DiD-labeled G-EVLPs).

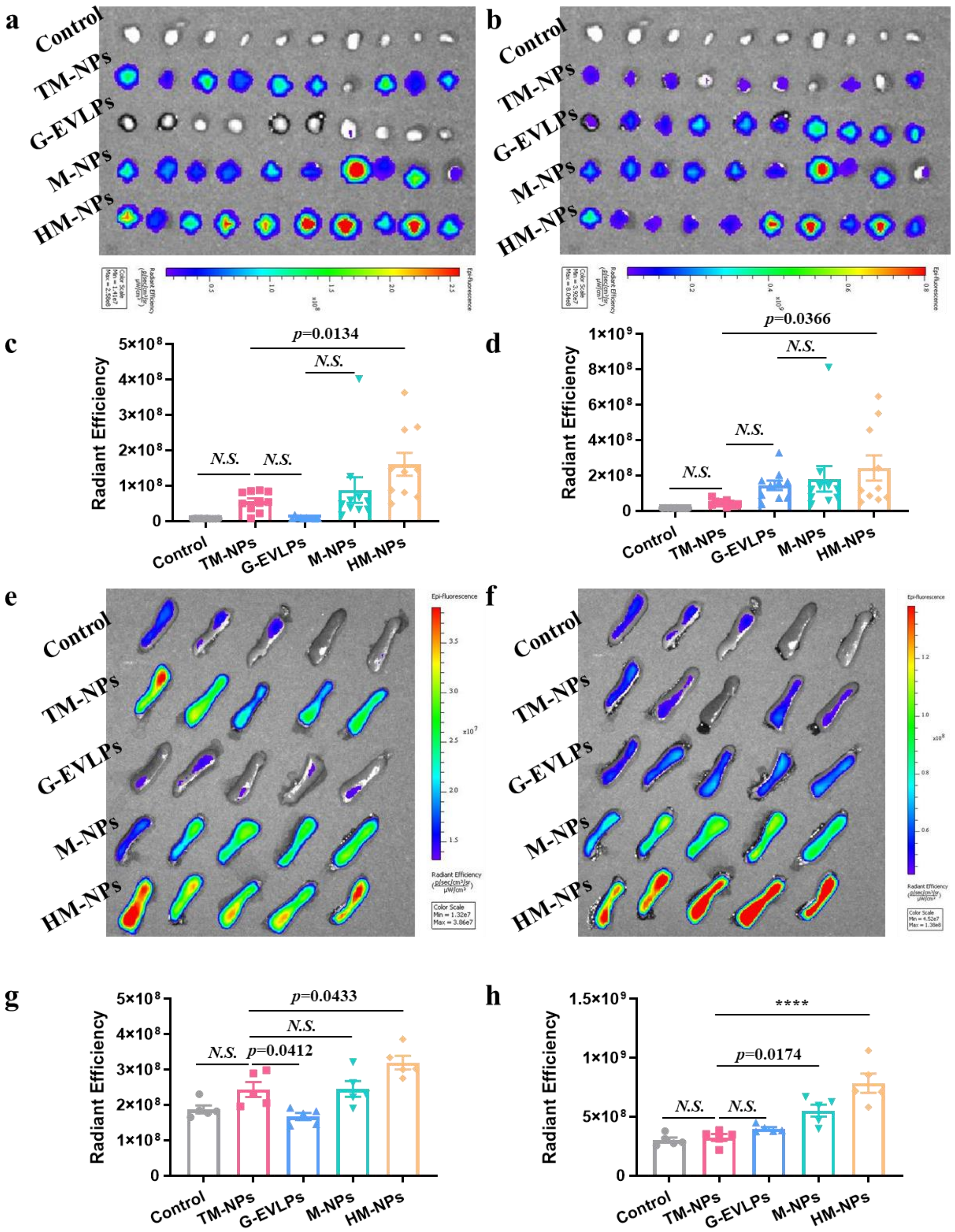

Figure S13. Distribution of different NPs in lymph nodes and spleens. a. Fluorescence images of LNs after vaccination (DiR-labeled tumor membrane). **b.** Fluorescence images of LNs after vaccination (DiD-labeled G-EVLPs). **c.** Fluorescence intensity analysis of inguinal lymph nodes after vaccination (DiR-labeled tumor membrane). **d.** Fluorescence intensity analysis of inguinal lymph nodes after vaccination (DiD-labeled G-EVLPs). **e.** Fluorescence images of spleens after vaccination (DiR-labeled tumor membrane). **f.** Fluorescence images of spleens after vaccination (DiD-labeled G-EVLPs). **g.** Fluorescence intensity analysis of spleens after vaccination (DiR-labeled tumor membrane). **h.** Fluorescence intensity analysis of spleens after

vaccination (DiD-labeled G-EVLPs). Data are representative or pooled and are expressed as Mean ± SE. Asterisks indicate statistically significant differences as analyzed by One-Way ANOVA (**** $p < 0.001$, N.S. $p > 0.05$).

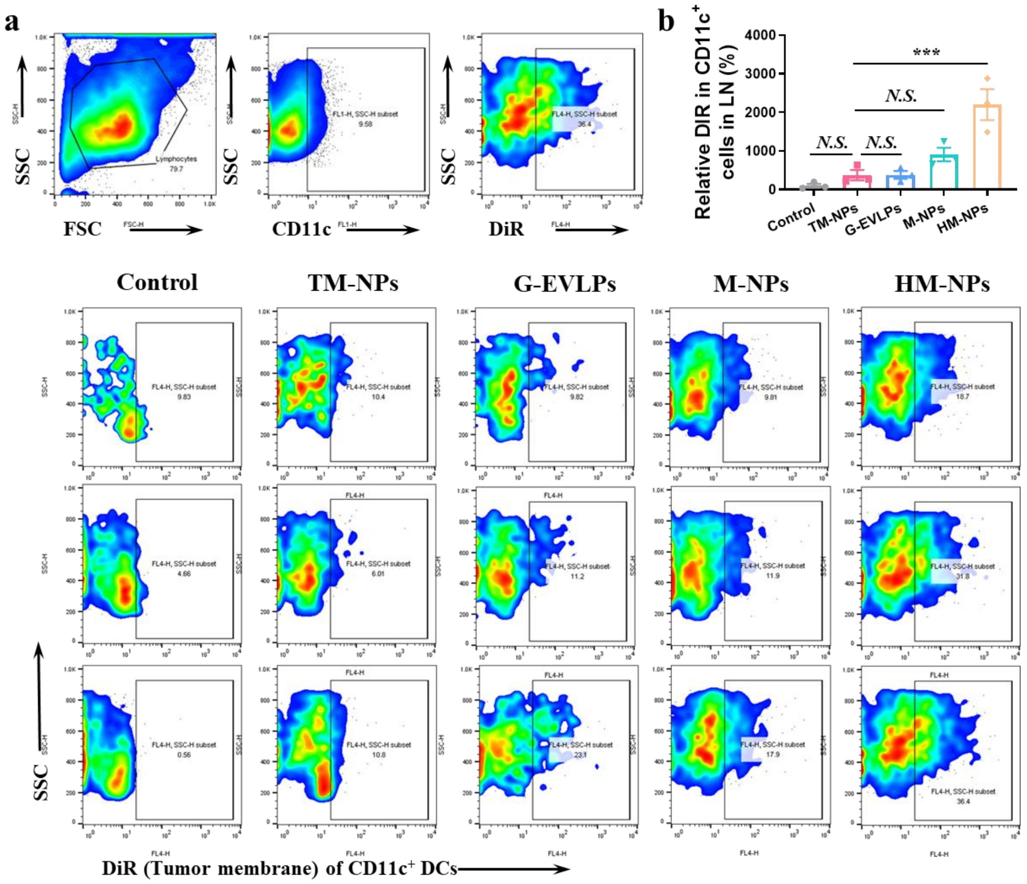

Figure S14. Flow cytometry analysis of DiD^{high} cells of CD11c^{+} DCs in inguinal lymph nodes after vaccination (DiR-labeled tumor membrane). a. Principles of flow cytometry data processing and flow cytometry uptake data for different groups. **b.** Relative cellular uptake of DiR-labeled tumor membrane of CD11c+ DC in inguinal lymph nodes, as assessed by flow cytometry.

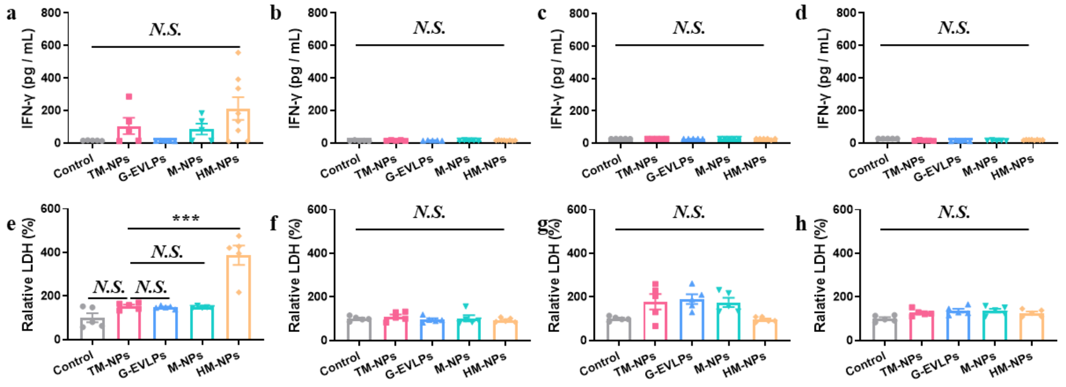

Figure S15. HM-NPs (B16F10) promote splenic T cells activation after vaccination. a-d. IFN-γ concentration in the supernatant after co-incubation of the splenic T lymphocytes with B16F10 tumor cells (a), CT26 tumor cells (b), MB49 tumor cells (c) and 4T1 tumor cells (d). **e-f.** LDH concentration in the supernatant after co-incubation of the splenic T lymphocytes with B16F10 tumor cells (e), CT26 tumor cells (f), MB49 tumor cells (g) and 4T1 tumor cells (h). Data are representative or pooled and are expressed as Mean ± SE. Asterisks indicate statistically significant differences as analyzed by One-Way ANOVA (*** p < 0.001, N.S. p>0.05).

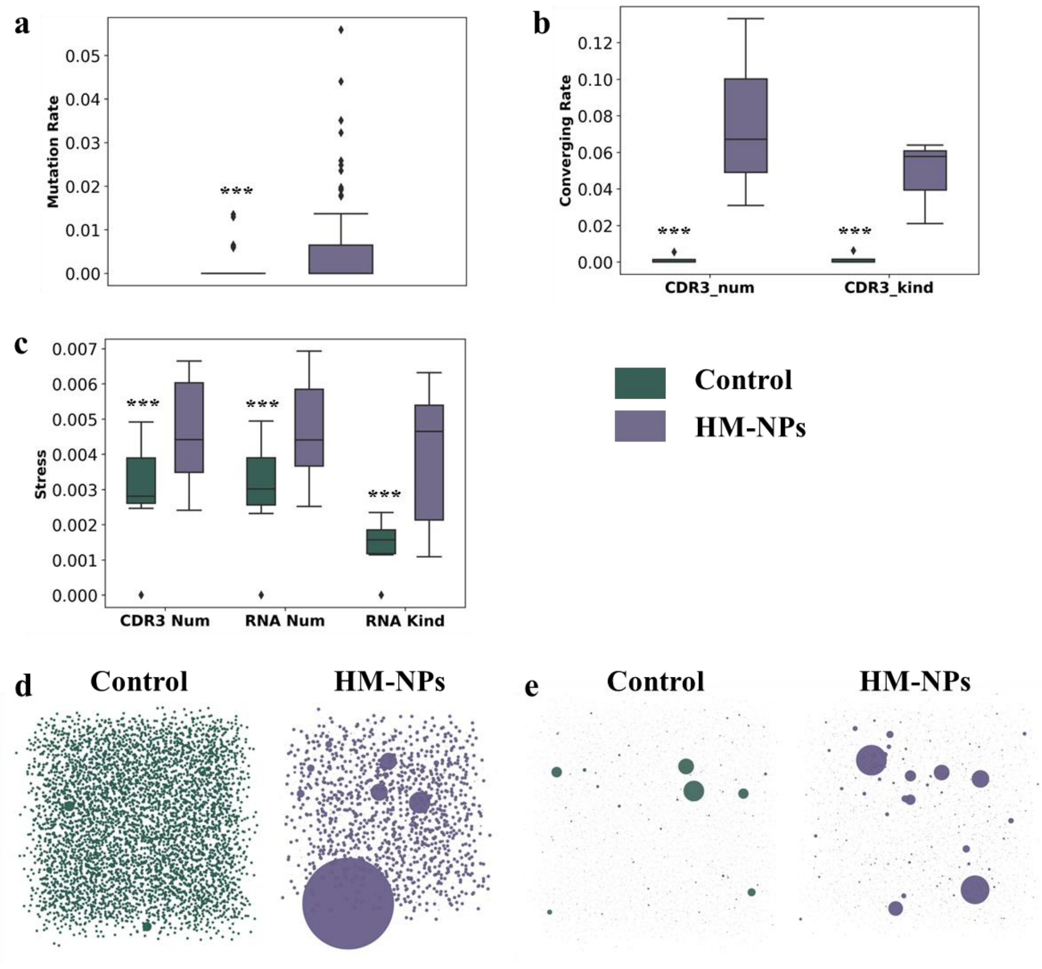

Figure S16. Repertoire analysis of splenic IgM B lymphocytes after vacation. a. The average mutation rate per base pair in the BCR V region. **b.** The comparison of the clone number and clone size distribution of CDR3 region reveals differences in the converging rate among different groups under the same RNA-UMI sequencing amount. **c.** At the levels of CDR3 clone number, RNA clone number, and unique RNA number, we calculate the extent to which the immune system diversity changed from control groups to HM-NPs. **d.** Visualizing the clone size distribution after clustering based on the CDR3 region similarity by Igraph package. **e.** Visualizing the clustering results that define clone size based on the number of UMIs linking to the same RNA, indicating the occurrence of polyclonal reactions. Data are representative or

pooled and are expressed as Mean ± SE (n=5). Asterisks indicate statistically significant differences as analyzed by One-Way ANOVA (*** $p < 0.001$).

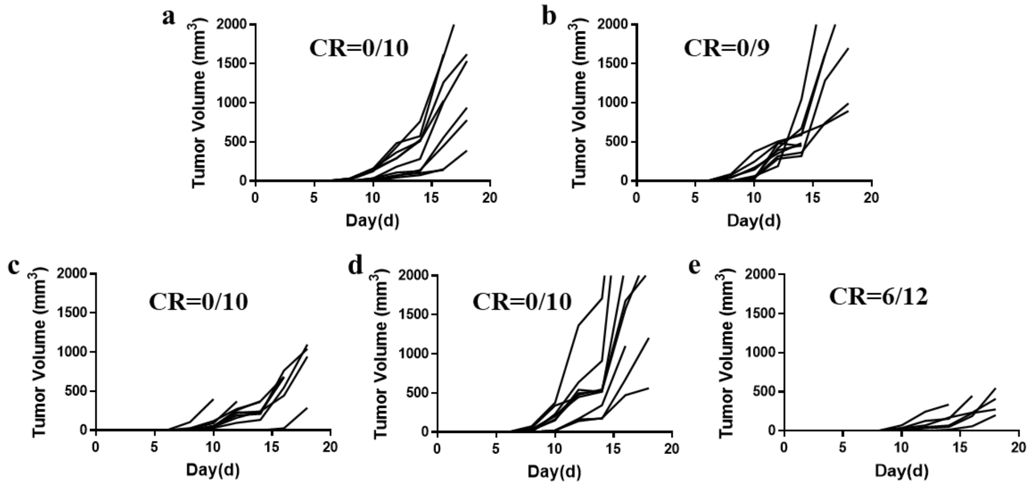

Figure S17. HM-NPs (B16F10) induce tumor recurrence suppression after vaccination. a-e. Tumor growth curves in the murine B16F10 tumor model of Control (a), TM-NPs (b), G-EVLPs (c), M-NPs (d) and HM-NPs (e).

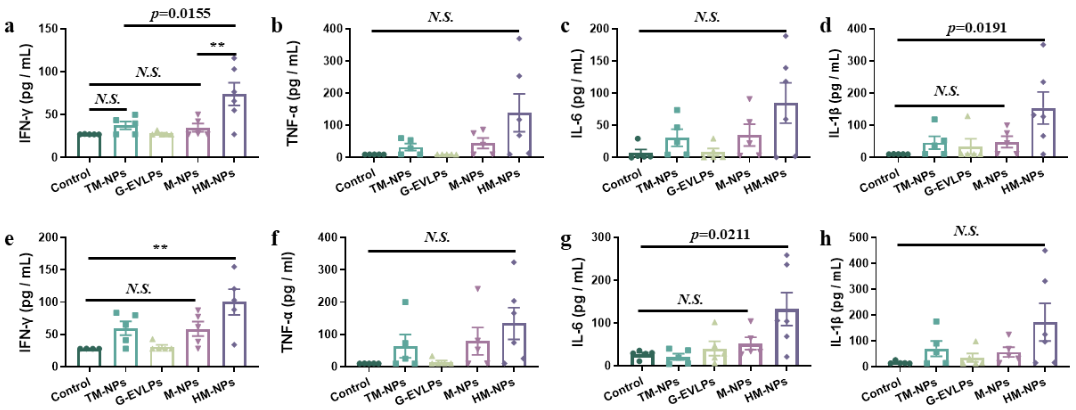

Figure S18. HM-NPs (B16F10) vaccination enhances anti-tumor immune response in the murine B16F10 tumor model. a. Proinflammatory IFN-γ concentration in the serum of the mice receiving each treatment determined by ELISA assay. **b.** Proinflammatory TNF-α concentration in the serum of the mice receiving each treatment determined by ELISA assay. **c.** Proinflammatory IL-6 concentration in the serum of the mice receiving each treatment determined by ELISA assay. **d.** Proinflammatory IL-1β concentration in the serum of the mice receiving each treatment determined by ELISA assay. **e.** Proinflammatory IFN-γ concentration in the tumor homogenate receiving each treatment determined by ELISA assay.

f. Proinflammatory TNF-α concentration in the tumor homogenate receiving each treatment determined by ELISA assay. **g.** Proinflammatory IL-6 concentration in the tumor homogenate receiving each treatment determined by ELISA assay. **h.** Proinflammatory IL-1β concentration in the tumor homogenate receiving each treatment determined by ELISA assay. Data are representative or pooled and are expressed as Mean ± SE. Asterisks indicate statistically significant differences as analyzed by One-Way ANOVA (** $p < 0.01$, N.S. $p>0.05$).

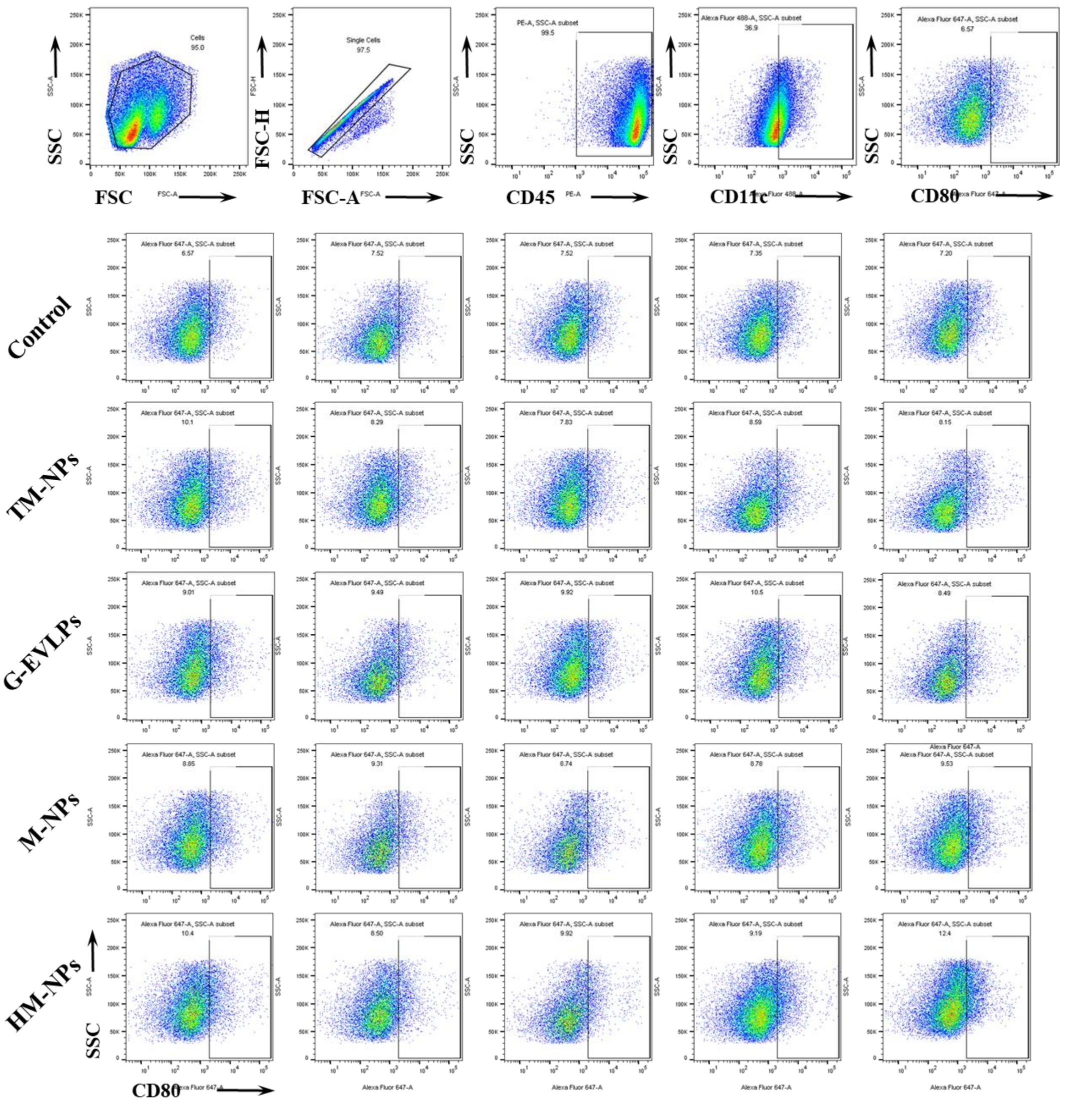

Figure S19. Flow cytometry analysis of CD45$^+$CD11c$^+$CD80$^+$ cells in LNs after vaccination.

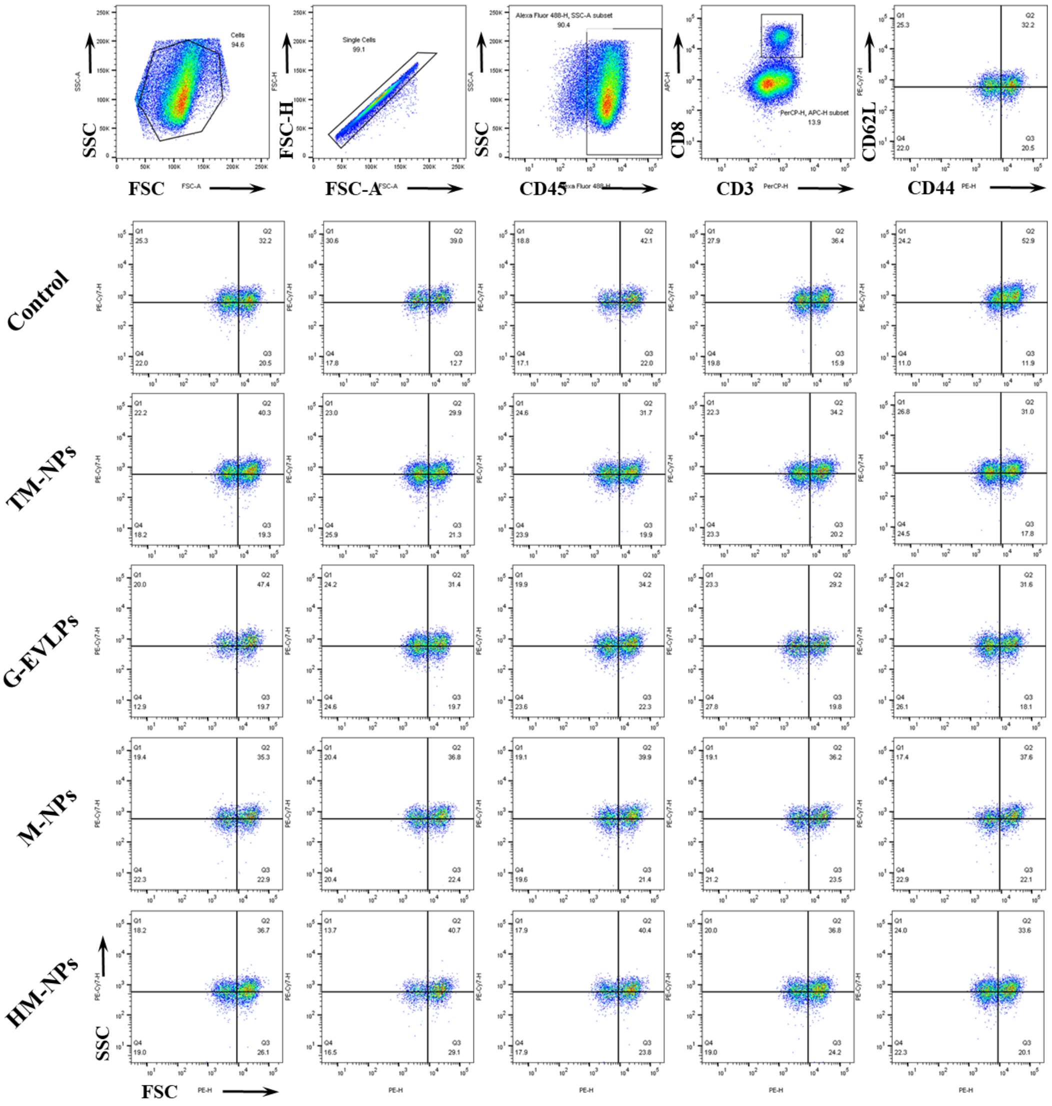

Figure S20. Flow cytometry analysis of immune memory cells in CD45$^+$CD3$^+$CD8$^+$ cells in spleen after vaccination.

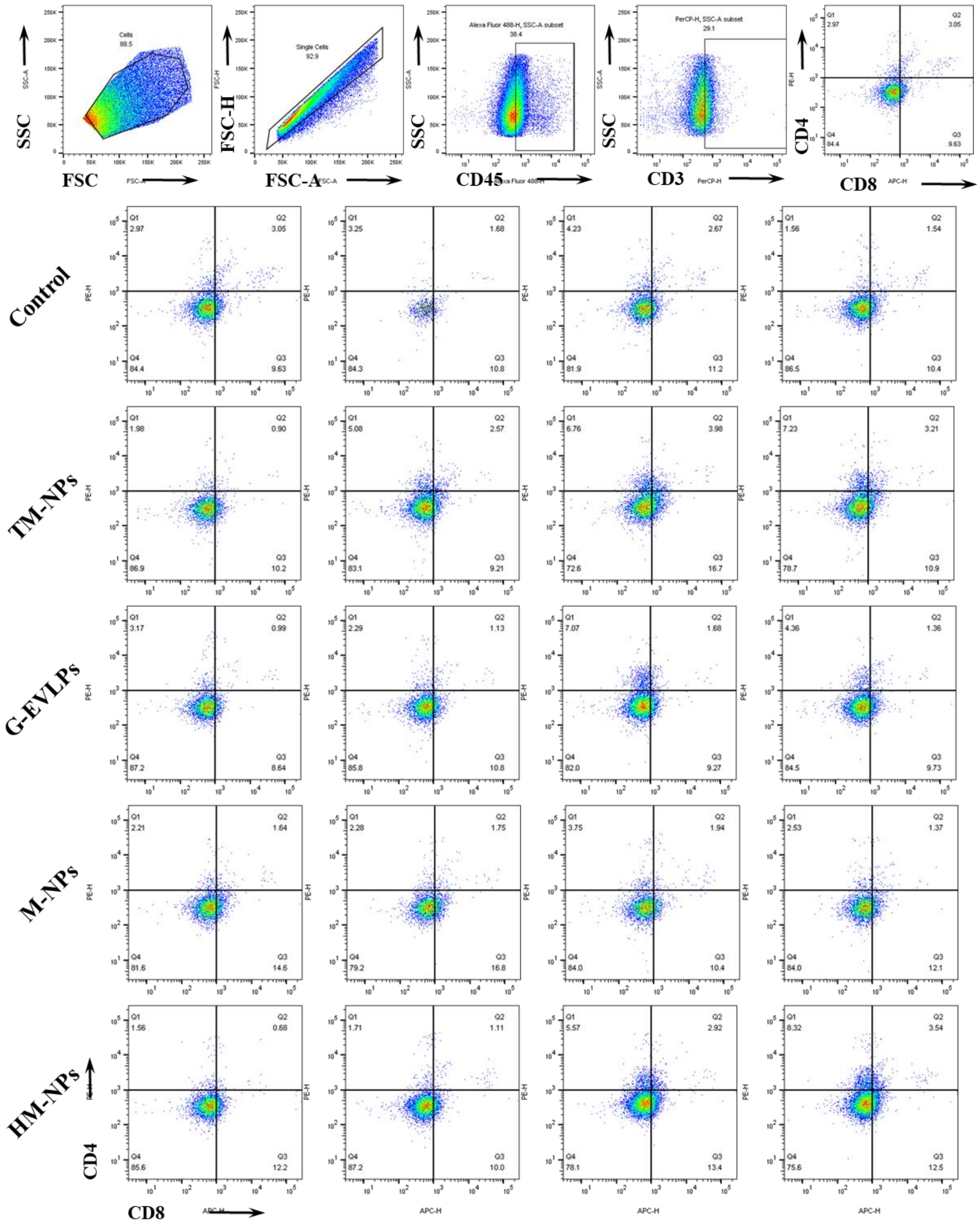

Figure S21. Flow cytometry analysis of CD45⁺CD3⁺CD4⁺ and CD45⁺CD3⁺CD8⁺ cells in tumors after vaccination.

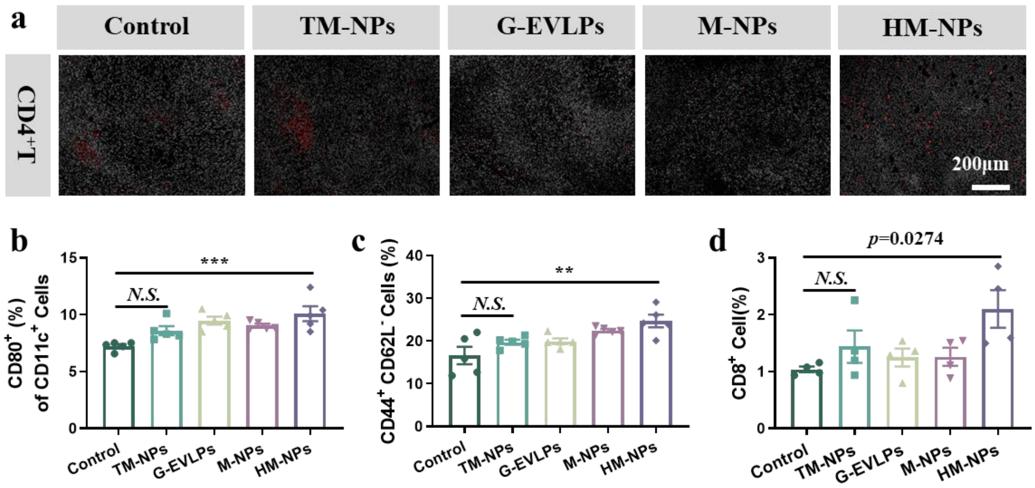

Figure S22. HM-NPs vaccination induces tumor recurrence suppression in the murine B16F10 tumor model. a. Immunofluorescence images of CD4$^+$ T of the tumors receiving different vaccinations. **b.** Flow cytometry analysis of CD11c$^+$ CD80$^+$ DCs in inguinal lymph nodes. **c.** Flow cytometry analysis of CD44^{high} CD62L^{low} (T_{EM}) DCs in inguinal lymph nodes. **d.** Flow cytometry analysis of CD45$^+$CD3$^+$ CD8$^+$ cells in tumors after vaccination. Data are representative or pooled and are expressed as Mean ± SE. Asterisks indicate statistically significant differences as analyzed by One-Way ANOVA (*** $p < 0.001$, ** $p < 0.01$, N.S. $p>0.05$).

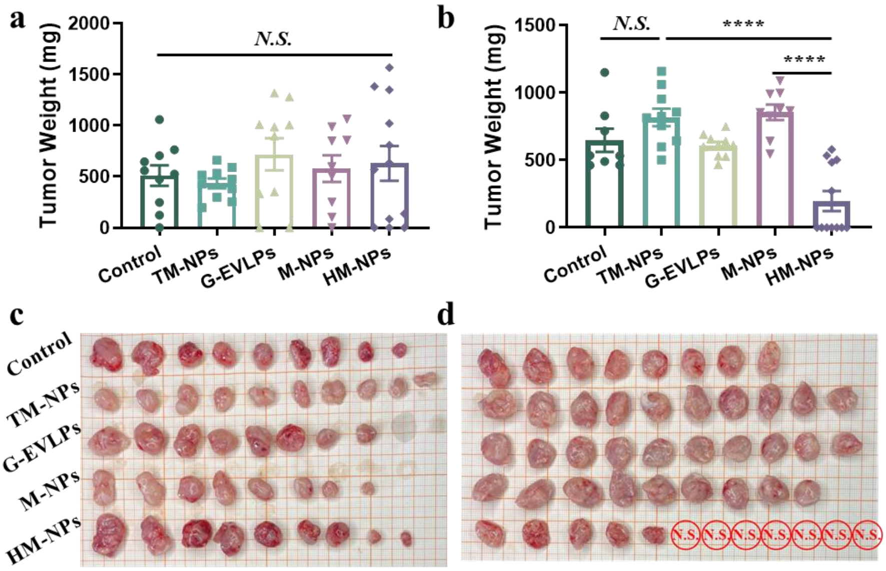

Figure S23. HM-NPs vaccination inhibits specific tumor recurrence. a. Tumor weight of each group in the murine CT26 tumor model at day 41. **b.** Tumor weight of each group in the murine 4T1 tumor model at day 41. **c.** Photographs of the murine CT26 tumor model at day 41. **d.** Photographs of the murine 4T1 tumor model at day 41. Data are representative or

pooled and are expressed as Mean ± SE. Asterisks indicate statistically significant differences as analyzed by One-Way ANOVA (**** $p < 0.001$, N.S. $p>0.05$).

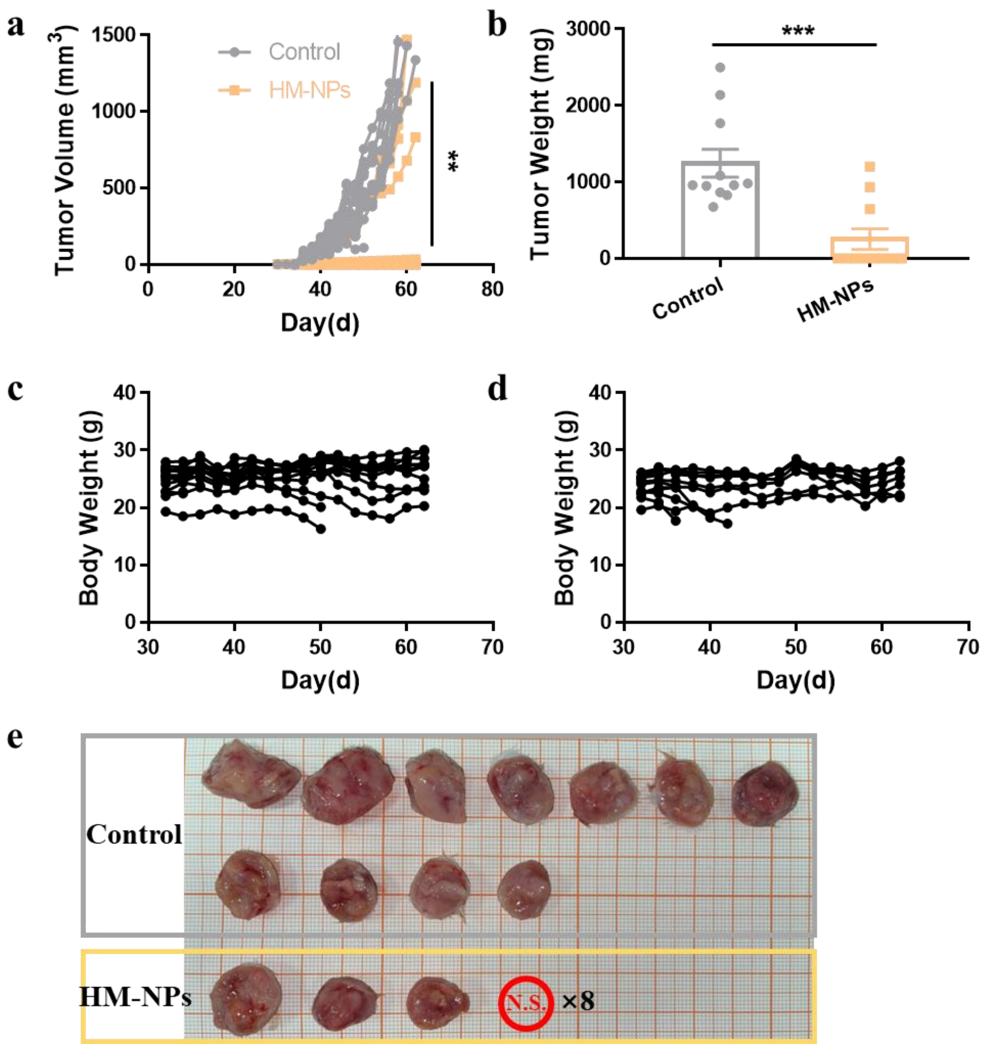

Figure S24. HM-NPs vaccination provides long-term anti-tumor protection. a. Tumor growth curves of each group in the murine 4T1 tumor model. **b.** Tumor weight of each group in the murine 4T1 tumor model at day 99. **c.** Body weight curves of the Control group in the murine 4T1 tumor model. **d.** Body weight curves of the HM-NPs group in the murine 4T1 tumor model. **e.** Photographs of the murine 4T1 tumor model at day 99. Data are representative or pooled and are expressed as Mean ± SE. Asterisks indicate statistically significant differences as analyzed by One-Way ANOVA (*** $p < 0.001$, ** $p < 0.01$)

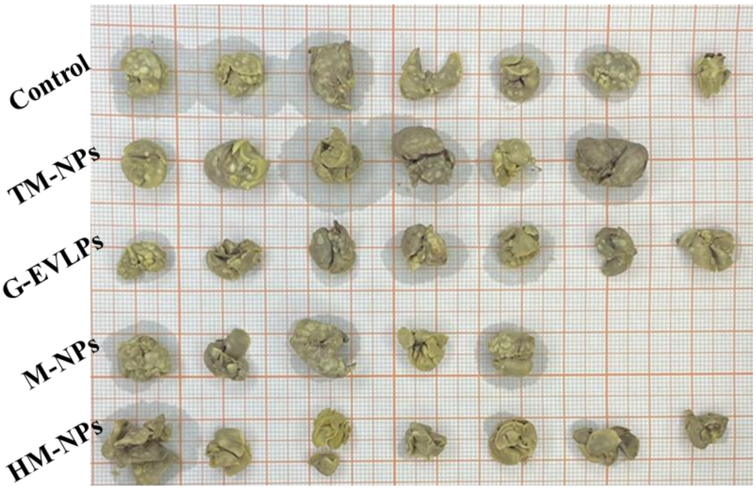

Figure S25. Photographs are the white filed images of lung tissues after staining with Bouin's Fluid.

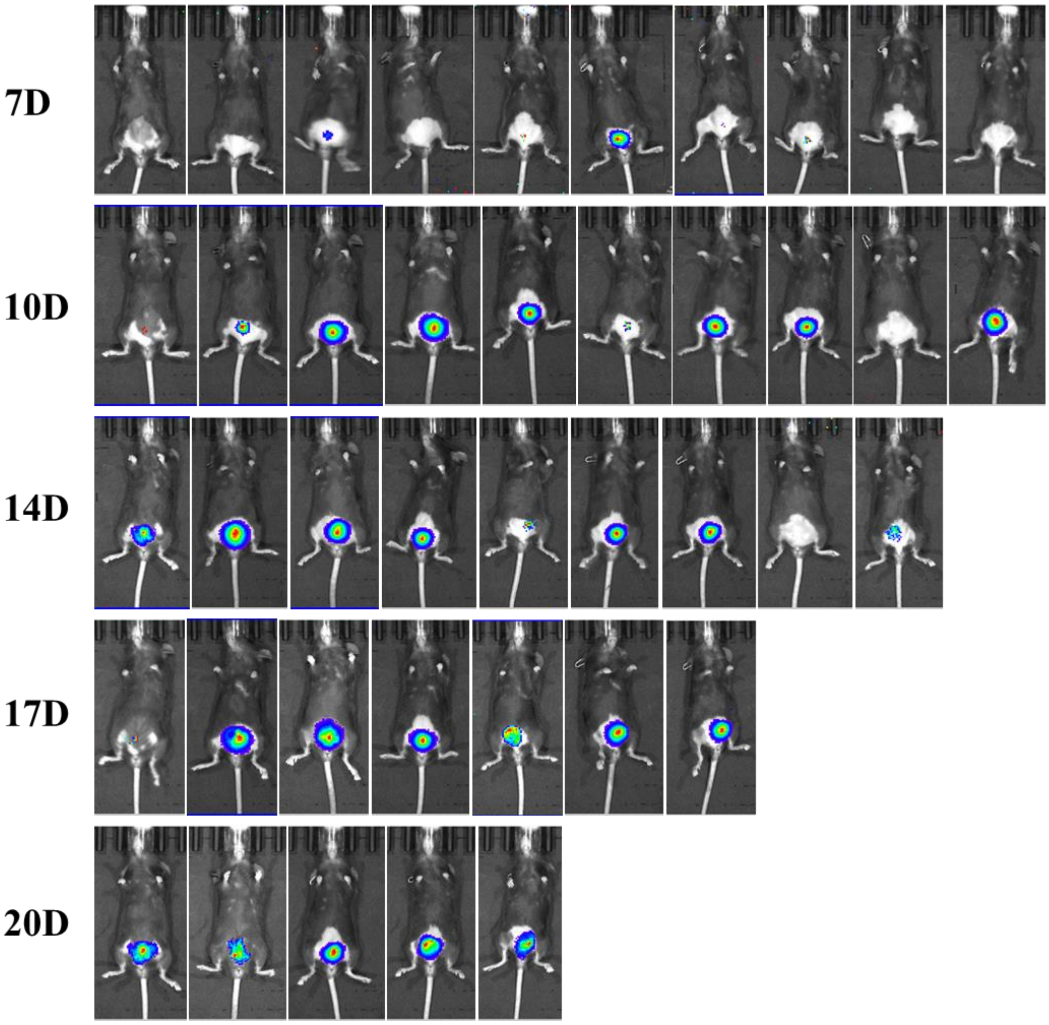

Figure S26. IVIS bioluminescence imaging of the mice after vaccination with Saline at days 7, 10, 14, 17 and 20 (post tumor re-inoculation).

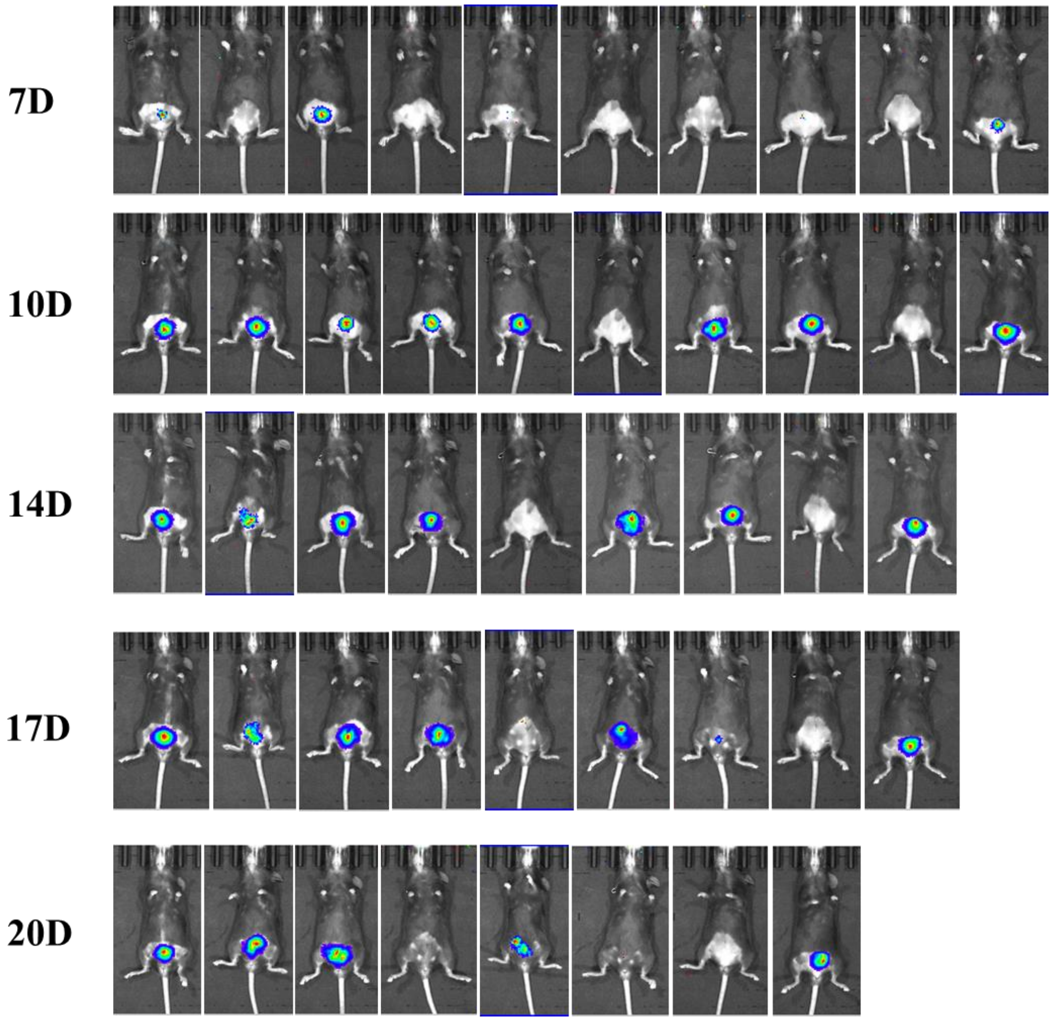

Figure S27. IVIS bioluminescence imaging of the mice after vaccination with TM-NPs at days 7, 10, 14, 17 and 20 (post tumor re-inoculation).

7D

10D

14D

17D

20D

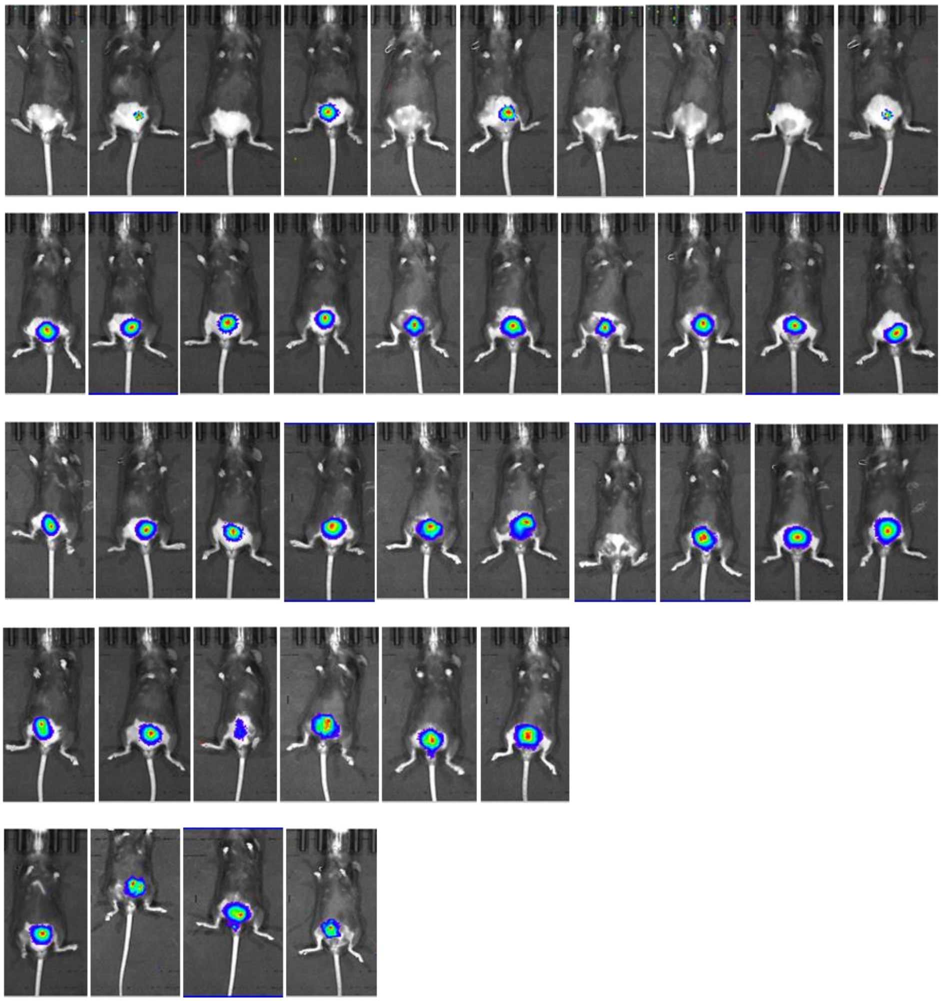

Figure S28. IVIS bioluminescence imaging of the mice after vaccination with G-EVLPs at days 7, 10, 14, 17 and 20 (post tumor re-inoculation).

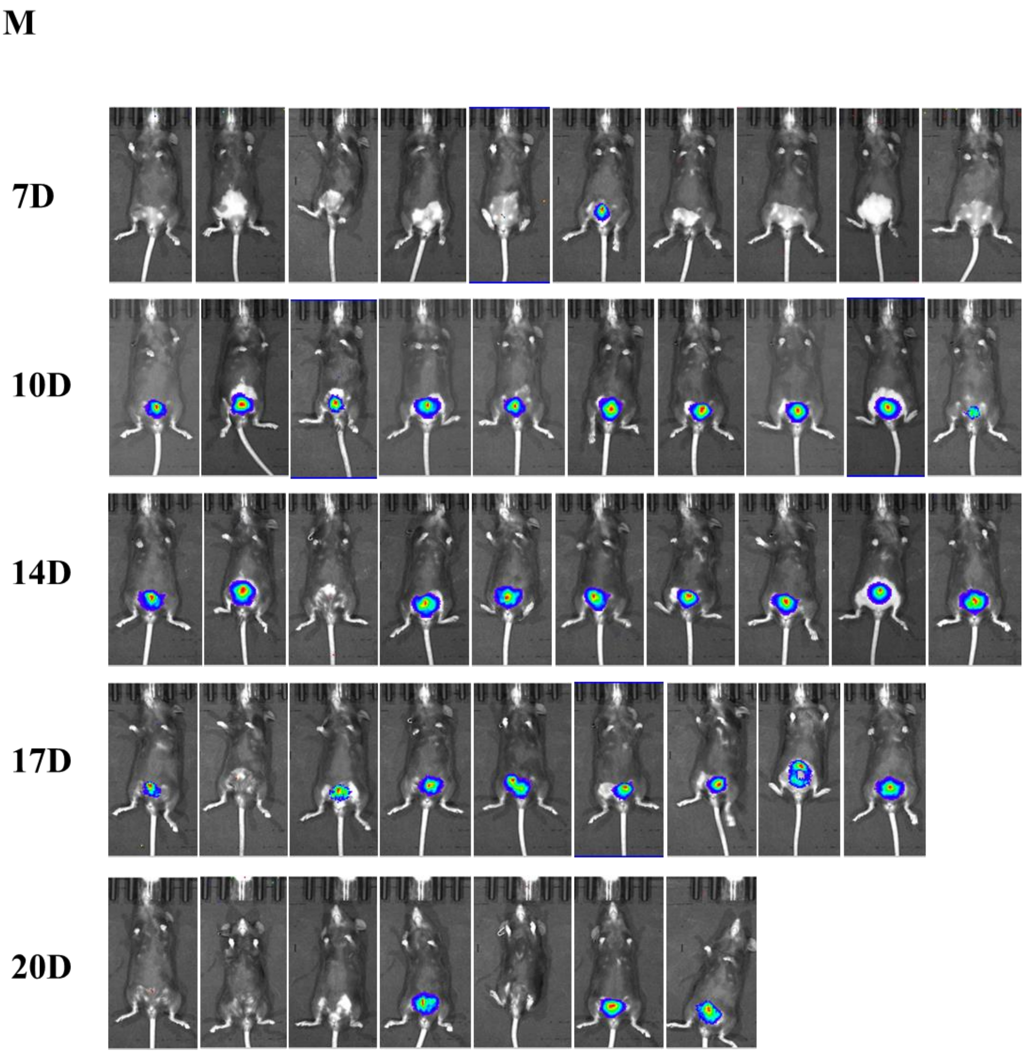

Figure S29. IVIS bioluminescence imaging of the mice after vaccination with M-NPs at days 7, 10, 14, 17 and 20 (post tumor re-inoculation).

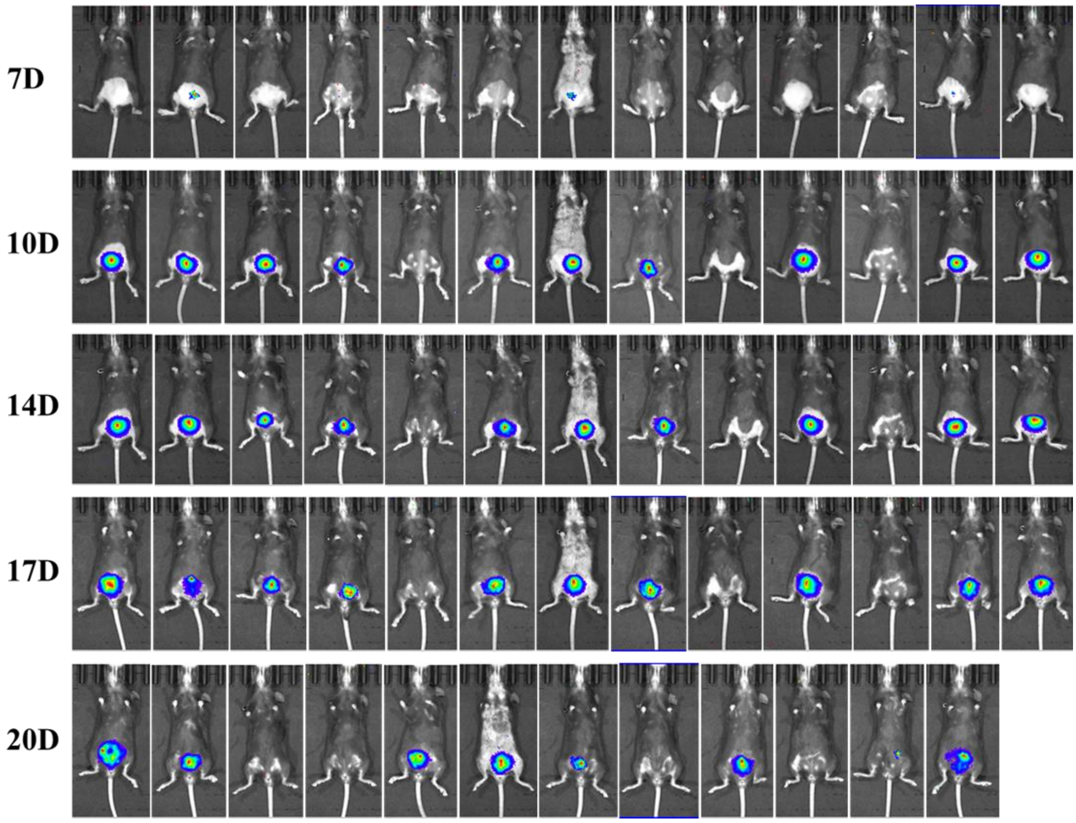

Figure S30. IVIS bioluminescence imaging of the mice after vaccination with HM-NPs at days 7, 10, 14, 17 and 20 (post tumor re-inoculation).

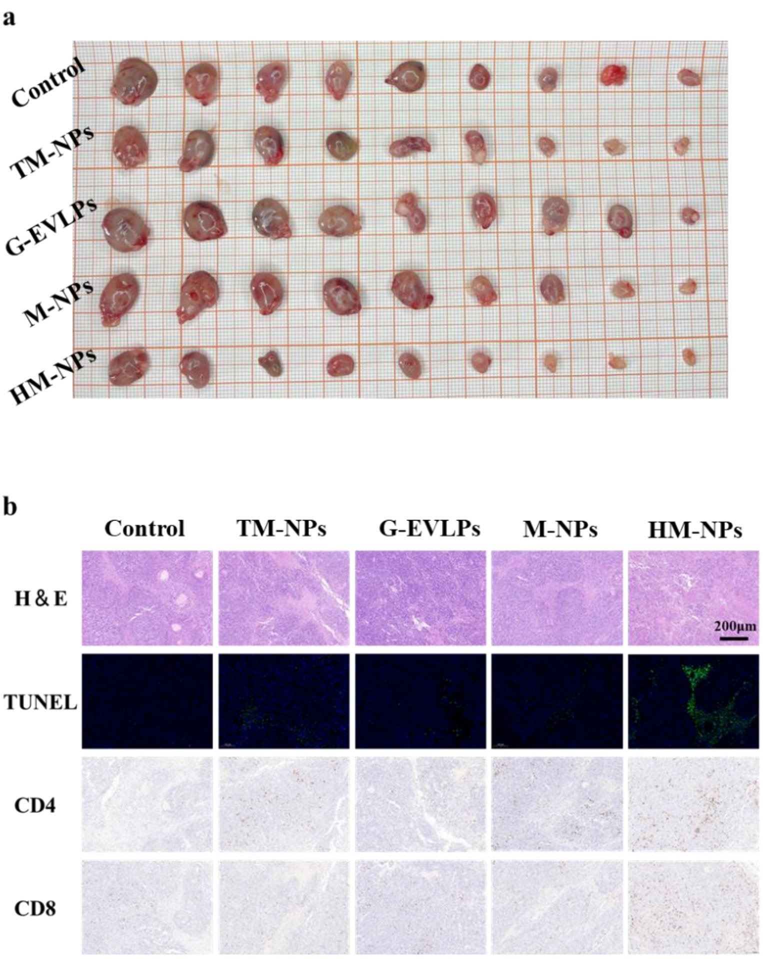

Figure S31. HM-NPs vaccination establishes protection against orthotopic mouse MB49 bladder tumor. a. Photographs of the murine MB49 tumor at day 36. **b.** Photographs are the H&E staining, TUNEL staining, and immunohistochemical images of the tumors post treatments of the tumors receiving different treatments

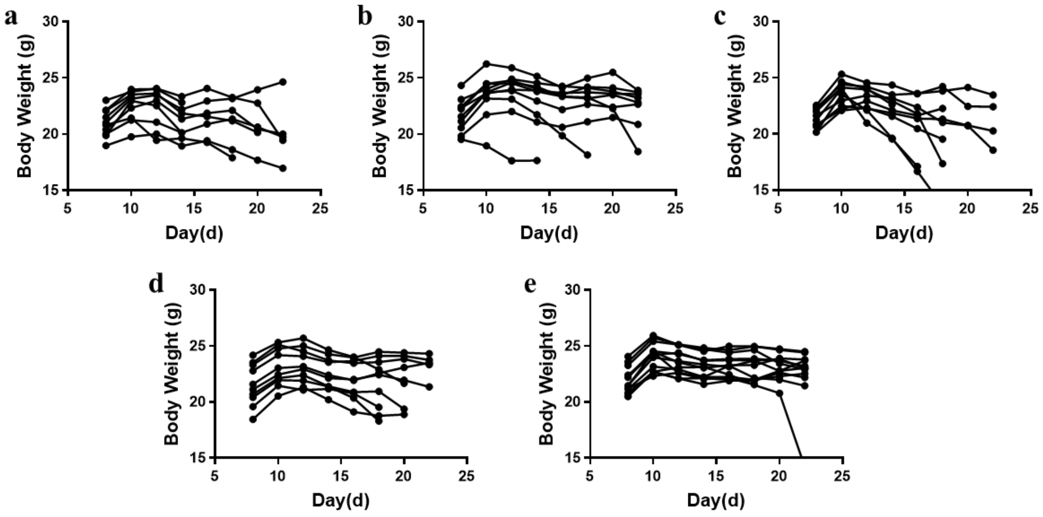

Figure S32. Body weight of mice after vaccination. a-e. Body weight curves in the murine MB49 tumor model of Control (a), TM-NPs (b), G-EVLPs (c), M-NPs (d) and HM-NPs (e).

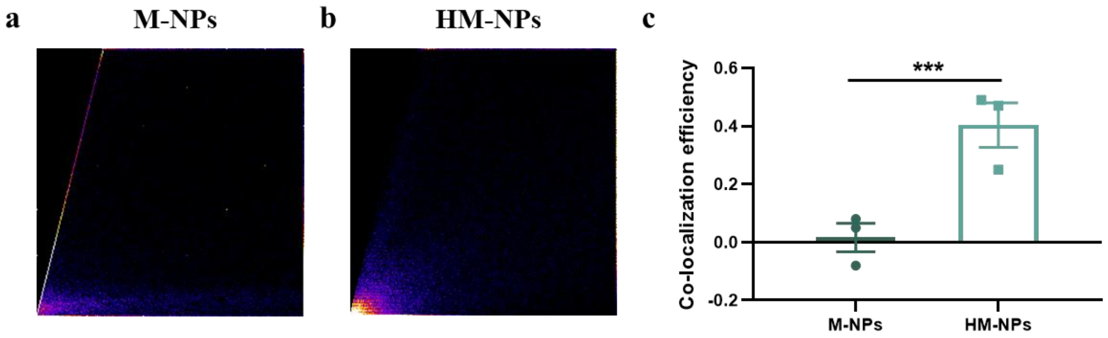

Figure S33. Localization of M-NPs and HM-NPs determined by CLSM. a. The Pearson Correlation coefficient of **M-NPs**. **b.** The Pearson Correlation coefficient of **HM-NPs**. **c.** The Pearson Correlation coefficient of **M-NPs and HM-NPs**. Data are representative or pooled and are expressed as Mean ± SE. Asterisks indicate statistically significant differences as analyzed by One-Way ANOVA (*** $p < 0.001$).

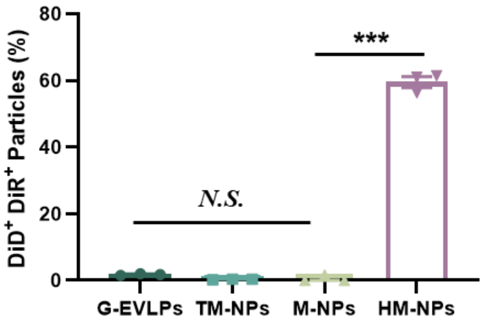

Figure S34. Analysis of the proportion of DiD and DiR double-positive particles using flow cytometry to determine the fusion efficiency of HM-NPs. Data are representative or pooled and are expressed as Mean ± SE. Asterisks indicate statistically significant differences as analyzed by One-Way ANOVA (*** $p < 0.001$).

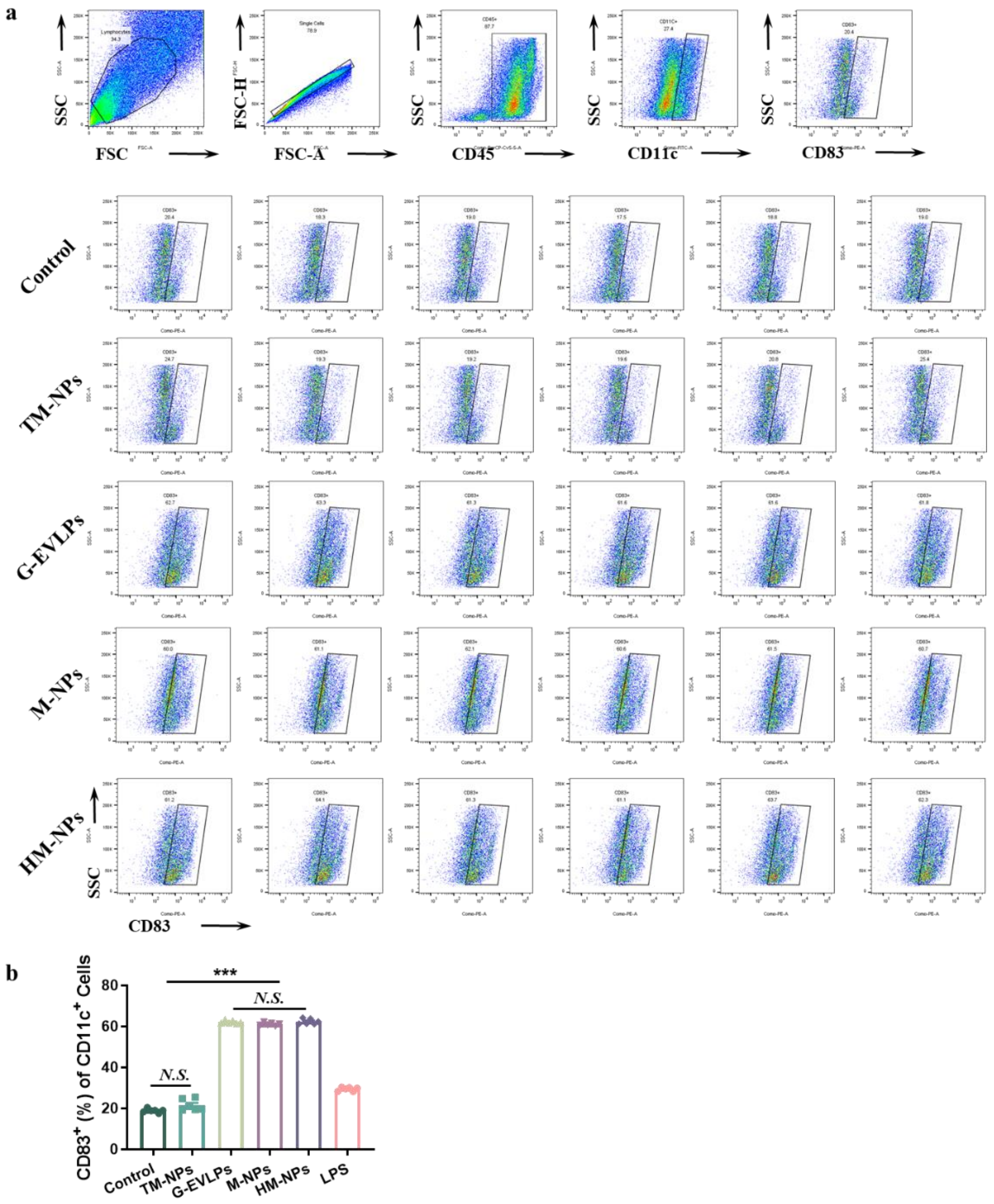

Figure S35. Flow cytometry analysis of CD45^{+}CD11c^{+}CD83^{+} cells in BMDCs. a. Figures of flow cytometry analysis**. b.** Statistical analysis of Figure **a.** Data are representative or pooled and are expressed as Mean ± SE. Asterisks indicate statistically significant differences as analyzed by One-Way ANOVA (*** $p < 0.001$).

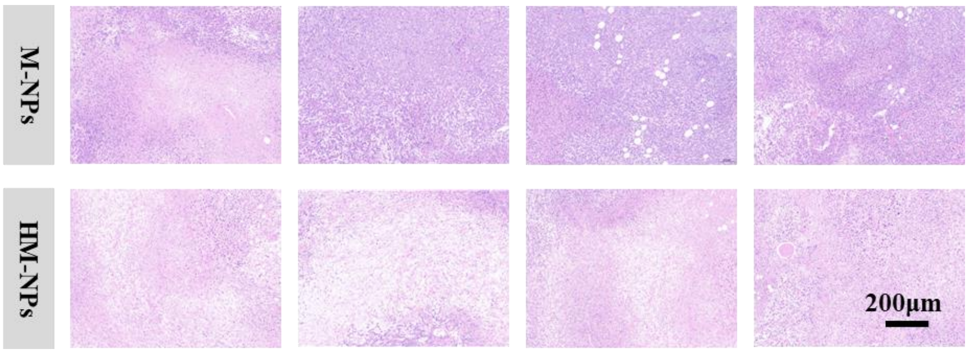

Figure S36. Photographs are the white filed images of the H&E staining of the tumors receiving different treatments.

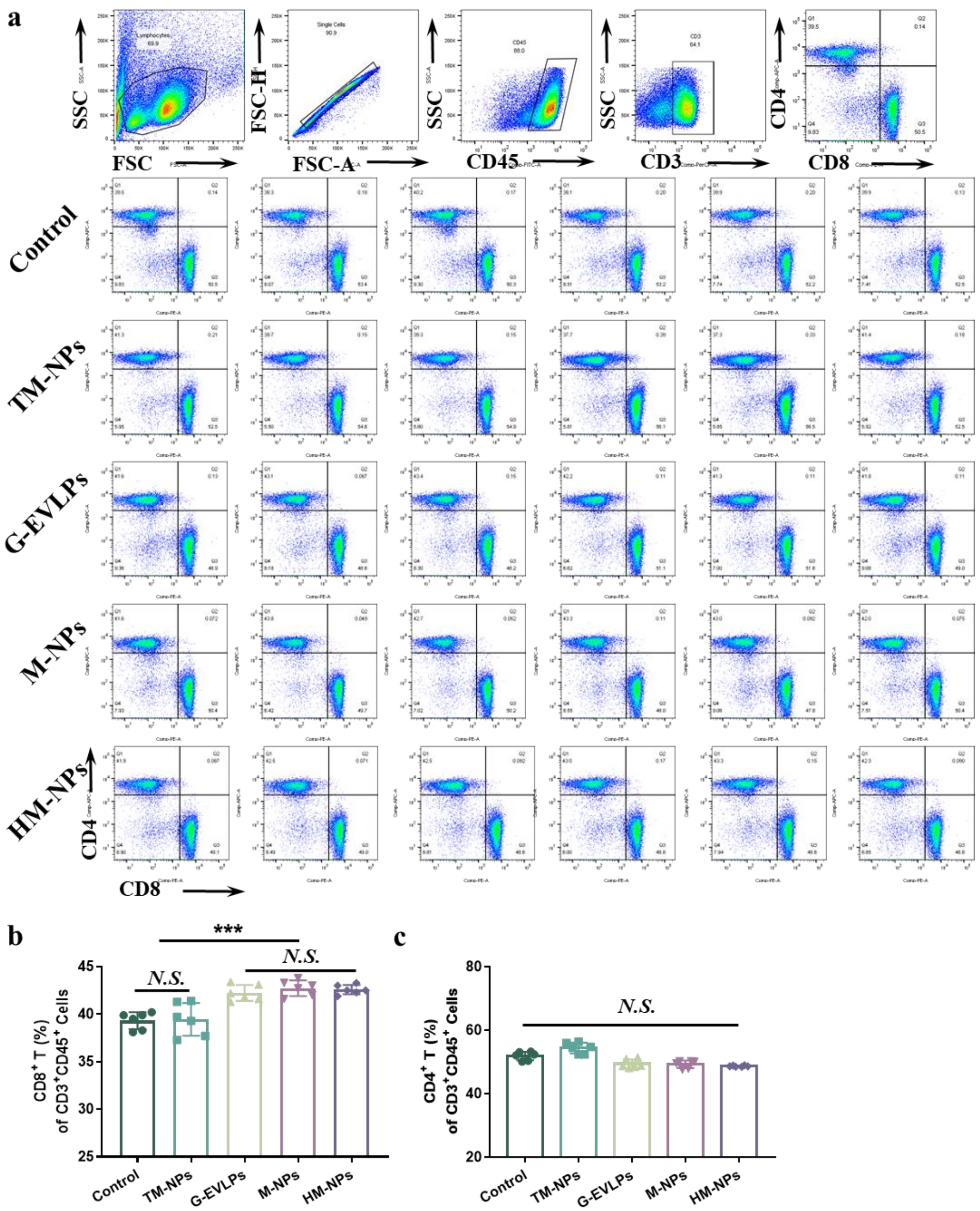

Figure S37. Flow cytometry analysis of $CD45^+CD3^+CD4^+$ and $CD45^+CD3^+CD8^+$ cells in LNs after vaccination. a. Flow cytometry analysis of $CD45^+CD3^+CD4^+$ and $CD45^+CD3^+CD8^+$ cells in LNs after vaccination. **b.** Statistical analysis of $CD45^+CD3^+CD8^+$ cells of Figure **a. c.** Statistical analysis of $CD45^+CD3^+CD4^+$ cells of Figure **a.** Data are representative or pooled and are expressed as Mean ± SE. Asterisks indicate statistically significant differences as analyzed by One-Way ANOVA (*** $p < 0.001$).

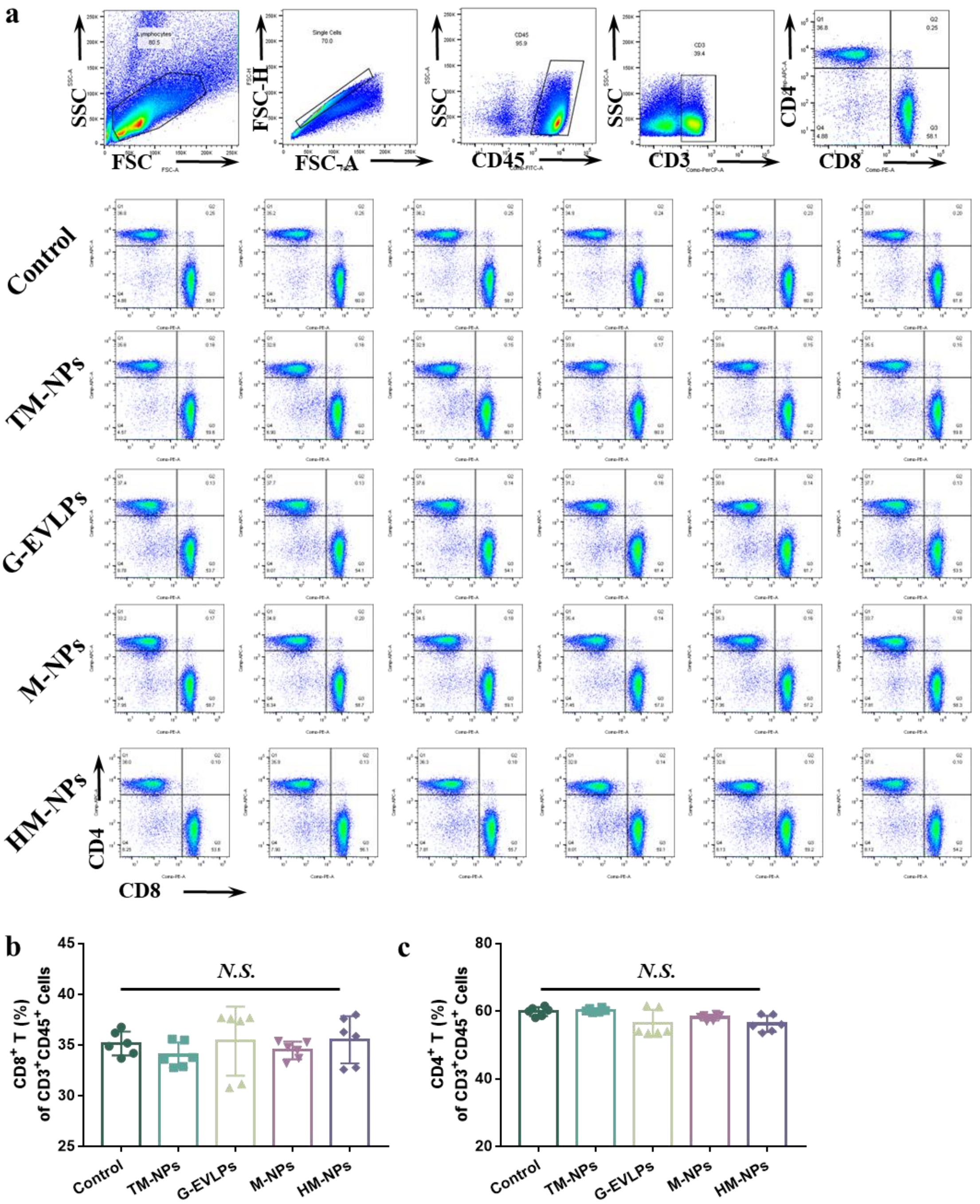

Figure S38. Flow cytometry analysis of CD45$^+$CD3$^+$CD4$^+$ and CD45$^+$CD3$^+$CD8$^+$ cells in Spleens after vaccination. a. Flow cytometry analysis of CD45$^+$CD3$^+$CD4$^+$ and CD45$^+$CD3$^+$CD8$^+$ cells in Spleens after vaccination. **b.** Statistical analysis of CD45$^+$CD3$^+$CD8$^+$ cells of Figure **a. c.** Statistical analysis of CD45$^+$CD3$^+$CD4$^+$ cells of Figure **a.** Data are representative or pooled and are expressed as Mean ± SE. Asterisks indicate statistically significant differences as analyzed by One-Way ANOVA.
